# Competition for nutritional resources masks the true frequency of bacterial mutants

**DOI:** 10.1186/s12915-020-00913-1

**Published:** 2020-12-14

**Authors:** Henrique Iglesias Neves, Gabriella Trombini Machado, Taíssa Cristina dos Santos Ramos, Hyun Mo Yang, Ezra Yagil, Beny Spira

**Affiliations:** 1 Departamento de Microbiologia, Instituto de Ciências Biomédicas Universidade de São Paulo, São Paulo, SP Brazil; 2 Departamento de Matemática Aplicada, Instituto de Matemática, Estatística e Computação Científica, Campinas, SP Brazil; 3 Departament of Biochemistry and Molecular Biology, Faculty of Life Sciences, Tel-Aviv University, Tel Aviv, Israel

**Keywords:** Mutagenesis, Mutation rate, Mutant frequency, Tragedy of the commons, PHO regulon, *pst* operon, Glycerol-2-phosphate

## Abstract

**Background:**

It is widely assumed that all mutant microorganisms present in a culture are able to grow and form colonies, provided that they express the features required for selection. Unlike wild-type *Escherichia coli*, PHO-constitutive mutants overexpress alkaline phosphatase and hence can hydrolyze glycerol-2-phosphate (G2P) to glycerol and form colonies on plates having G2P as the sole carbon source. These mutations mostly occur in the *pst* operon. However, the frequency of PHO-constitutive colonies on the G2P selective plate is exceptionally low.

**Results:**

We show that the rate in which spontaneous PHO-constitutive mutations emerge is about 8.0 × 10^−6^/generation, a relatively high rate, but the growth of most existing mutants is inhibited by their neighboring wild-type cells. This inhibition is elicited only by non-mutant viable bacteria that can take up and metabolize glycerol formed by the mutants. Evidence indicates that the few mutants that do form colonies derive from microclusters of mutants on the selective plate. A mathematical model that describes the fate of the wild-type and mutant populations under these circumstances supports these results.

**Conclusion:**

This scenario in which neither the wild-type nor the majority of the mutants are able to grow resembles an unavoidable “tragedy of the commons” case which results in the collapse of the majority of the population. Cooperation between rare adjacent mutants enables them to overcome the competition and eventually form mutant colonies. The inhibition of PHO-constitutive mutants provides an example of mutant frequency masked by orders of magnitude due to a competition between mutants and their ancestral wild-type cells. Similar “tragedy of the commons-like” cases may occur in other settings and should be taken into consideration while estimating true mutant frequencies and mutation rates.

**Supplementary Information:**

The online version contains supplementary material available at (doi:10.1186/s12915-020-00913-1).

## Background

The frequency of mutants in a population is central to the understanding of evolution. The rate at which mutations occur is characteristic of the species, but it is also shaped by environmental conditions [1–3]. Under stressful conditions, a fraction or the entire bacterial population increase the mutation rate by some amount in what appears to be a response mechanism to environmental challenges [3–5]. Another factor that may affect the assessment of mutation rate/mutant frequency is the density of the bacterial population in the selective medium [6–8]. For instance, traces of usable nutrients may foment bacterial replication on the plate and falsely increase the number of mutant colonies [7, 8]. This especially occurs in mutational systems in which the selective plates are incubated for longer periods of time, usually more than 48 h, during which the bacterial population grows slowly using non-selective alternative nutrient traces. For this reason, it is a usual practice to plate, together with the test bacteria, “scavenger” or “filler” cells that cannot mutate to prototrophy, but can scavenge nutrient traces from the medium [6, 7, 9]. On the other hand, *Escherichia coli* cultures grown to high densities have consistently shown lower rifampicin resistance mutation rates, in a mechanism dependent on cell–cell signaling and on the quorum sensing-related gene *luxS* [10]. This finding was recently extended to the yeast *Saccharomyces cereviseae*, in which an inverse relation between cell density and mutation rate has been also observed [11]. Thus, while addition of filler cells is necessary to prevent overestimation of mutant frequency in non-lethal selection systems, high cellular concentration may lead to an underestimation of mutant frequency due to density-associated mutation inhibition.

The PHO regulon of *E. coli* comprises more than 30 genes associated with the uptake and assimilation of orthophosphate (Pi)-containing molecules [12]. Genes belonging to the PHO regulon are controlled by the two-component system PhoB/PhoR, being repressed under Pi-excess and induced under conditions of Pi-deprivation. The signal about Pi availability in *E. coli*’s periplasm is transferred to the histidine kinase PhoR via the intermediation of Pi-specific transport (Pst) system. Pst is encoded by the *pstSCAB-phoU* genes, or in short *pst* operon. Mutations in any one of the five *pst* operon genes result in the constitutive expression of the PHO regulon, i.e., maximal synthesis of all PHO proteins even under conditions of excess Pi [13, 14]. Thus, the Pst system, besides being the principal Pi-uptake system in *E. coli*, also acts as a repressor of the PHO regulon [15–17].

PHO-constitutive *pst* mutant colonies can be selected on minimal medium plates containing glycerol-2-phosphate (G2P) as the sole carbon (C) source and excess Pi [13]. *E. coli* cannot utilize G2P as a C source unless it is cleaved to glycerol and Pi by the periplasmic, *phoA*-encoded enzyme alkaline phosphatase (AP), another PHO-regulon member. The glycerol moieties released by AP activity thereby enter the cell mostly by facilitated diffusion via GlpF [18, 19]. Thus, the constitutive expression of AP caused by the *pst* mutation enables *E. coli* to grow on G2P medium [14, 20]. It should be added that some mutations in *phoR* also result in the constitutive expression of the PHO regulon and likewise confer the ability to grow on G2P. However, all PHO-constitutive mutations (PCMs) derived from strain MG1655 hitherto analyzed were in the *pst* operon [14].

We have previously shown that when ∼10^9^ bacteria are plated on G2P plates as a sole C source, only around 100 PHO-constitutive colonies emerge on the selective plate, a mutant frequency of 10^−7^. That is despite the relatively large 5-kb target for PHO-constitutive mutations (five *pst* operon genes and a few bases in *phoR*). In addition, the first PCM colonies appear only after 3 days of incubation, and their number on the selective plates keeps increasing with time in a sigmoid pattern [14]. The observation that PCMs, once purified, form colonies on G2P plates within less than 48 h led us to suggest that the late emergence of PCMs on the selective medium could be explained by stress-induced adaptive mutagenesis [2, 14]. In further investigations reported below, we demonstrate that the conundrum of low mutant frequency and late emergence of mutant colonies is caused by PCM growth inhibition in a high-density population. The glycerol produced by the PCMs leaches out and is sequestered by the overwhelming presence of surrounding wild-type cells. Since the concentration of glycerol is not sufficient to support the growth of both PCM and wild-type cells, the population collapses, as in a “tragedy of the commons-like” case [21–23]. Only a tiny fraction of the PCMs eventually manage to grow and form colonies. These colonies originate from clusters of PCM cells that through mutual feeding of glycerol acquire a slight advantage over the wild-type cells that only consume but do not produce glycerol.

## Results

### Characterization of the emerging PHO-constitutive mutants

**Fig. 1.**
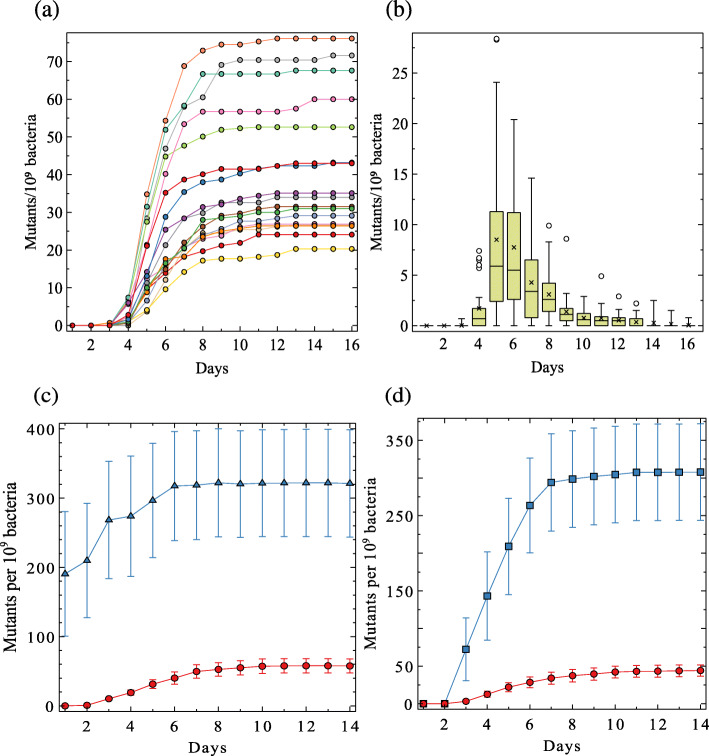
**a** Daily accumulation and distribution of PCMs in 17 independent cultures. 10^9^ MG1655 bacteria were plated on each TG2PP plate. The number of mutants emerging on the selective plates is recorded for 16 days. **b** Boxplots representing the distribution of new colonies appearing on each day in the 17 plates. (x), (-) and () represent the means, medians, and outliers, respectively. **c** Frequency of PCMs and rifampicin-resistant mutants. 10^9^ bacteria (MG1655) were plated on TGP minimal medium supplemented with rifampicin (100 µg/ml) or on TG2PP plates. , PCMs; , Rif^R^ mutants. Each point represents the mean ± S.E.M. of 10 independent cultures. **d** Selection of PCMs in a *mutS* background. 10^9^ MG1655 or *ΔmutS*::Cm bacteria were each separately plated on TG2PP plates. , PCMs from MG1655; , PCMs from *mutS*. Each point represents the mean ± S.E.M. of seven independent cultures

The pattern of PCM emergence in a population of *E. coli* cells was followed. An overnight culture containing 10^9^ wild-type MG1655 cells is plated on TG2PP-minimal medium containing G2P as the sole carbon source, excess Pi, and the AP substrate XP that stains the constitutive mutants blue owing to their high AP activity. Figure 1a shows the kinetics of PCM accumulation in 17 such independent cultures. In all of them, the first few colonies emerged on the 3rd–4th day of incubation, while more colonies kept appearing for the next 10 days, with the bulk of colonies emerging between the 5th and the 8th day following plating. The mean number of mutant colonies at the end of 16 days was 41.1±17.9. Typically, the mutant frequency oscillates between 20 and 200 mutants/plate (0.2−2.0×10^−7^ mutants/cell/generation). The distribution of PCM emergence per day is shown in Fig. 1b. The average mutant frequency in *E. coli* is between 10^−6^ and 10^−7^ per gene [24–26]. Thus, the expected mutant frequency for mutations in the five *pst* operon genes should have been 5×10^−7^ to 5×10^−6^/cell/generation, roughly 25–250-fold higher than the frequency observed here.

To test whether other types of mutants also display delayed appearance and low frequency on medium TGP, we assessed the frequency of rifampicin-resistant mutants (Rif^R^) in strain MG1655. Overnight grown bacteria were plated on medium TGP containing rifampicin (100 µg/ml) or on TG2PP plates (Fig. 1c). Sixty percent of the Rif^R^ colonies were already present 24 h following plating, while new colonies kept emerging up to day 6. The mutant frequency at the end of the experiment was 3.2×10^−7^/generation, which is compatible with data described elsewhere for strain MG1655 [27, 28], but unlike others that reported lower frequencies (∼2.0×10^−8^/generation) [29, 30]. In contrast, bacteria plated on TG2PP showed the usual pattern of mutant accumulation (first visible colonies at day 3 and a low mutant frequency of 5.8×10^−8^/generation, calculated at the 14th day). The high frequency of Rif^R^ colonies that were evident in the first 3 days indicates preexisting spontaneous mutations in the overnight cultures. By comparison, on average, only 17% of the PCM colonies emerged on the selective plates at day 3 and additional ones kept appearing up to day 10. It is worth mentioning that upon restreaking on G2P plates even the late PCMs form colonies within 48 h. The late Rif^R^ colonies, which emerged from the 3rd day onward, were probably slow-growing mutants [31, 32].

To test whether the low PCM frequency and their late appearance could be relieved by a mutator phenotype, we assessed the emergence of PCMs in strain MG1655 *ΔmutS* (strain RI103). The *ΔmutS* knockout is partially deficient in DNA mismatch repair and displays a 10–100 fold increase in mutation rate [33–35]. Figure 1d shows that the *mutS* strain accumulated 7.5 times more PCM colonies than its *mutS*^+^ parent at the end of the experiment at day 14th. However, no colony emerged before the 3rd day of incubation and the sigmoid pattern of mutant accumulation was similar to the one observed in the wild-type strain, suggesting that the late emergence pattern of PCMs is not necessarily associated with the low frequency of these mutants.

The late emergence and low frequency of PCMs may hint that these mutants were not pre-existent, but appear only after the bacteria are exposed to the selective conditions (G2P as the C source). Another possibility is that the PCMs are present in the pre-culture (prior to their contact with G2P), but that for some reason their growth on the selective plate is being inhibited. The following experiments were aimed at investigating this second hypothesis.

**Fig. 2.**
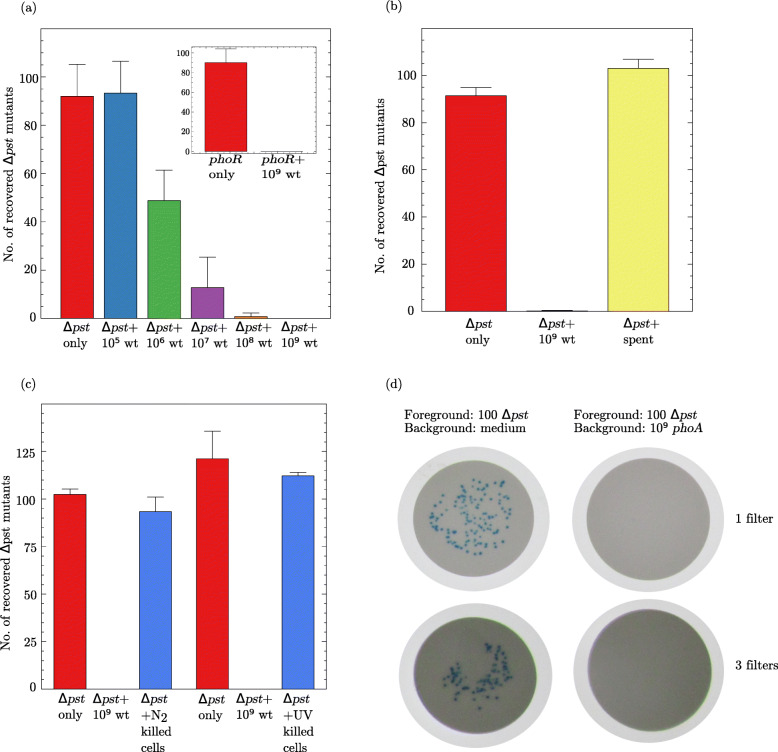
**a** Growth inhibition of ∼100 *Δpst*::Km cells plated with increasing amounts of wild-type MG1655 bacteria (wt). Inset shows the growth of *phoR* cells plated with and without 10^9^ wild-type cells. Each bar represents the mean ± S.E.M. of 5 independent experiments. **b** Effect of wild-type spent medium on PCM growth. 10^9^ MG1655 cells grown overnight in medium TGP were resuspended in TG2PP and further incubated at 37 ^∘^C for 48 h. The filtered supernatant of this culture (spent) was mixed with 100 *Δpst*::Km cells and plated on TG2PP for another 48 h (*Δpst* + spent). One hundred *Δpst*::Km (*Δpst* only) and 100 *Δpst*::Km mixed with 10^9^ wild-type cells (*Δpst* + 10^9^ wt) were plated as controls. **c** Growth inhibition by dead cells. 10^9^ wild-type bacteria were killed either by freeze-thawing in liquid nitrogen (N_2_ killed cells) or by UV-irradiation (UV killed cells). The dead bacteria were mixed with 100 *Δpst* cells, plated on TG2PP and incubated for 48 h. *Δpst* cells alone and *Δpst* cells mixed with 10^9^ untreated wild-type cells served as controls. Each bar represents the mean ± S.E.M. of 5 independent cultures. **d** Growth inhibition of *Δpst* PCMs does not require contact with inhibitor cells. *Δpst*::Km cells were grown overnight in medium TGP, washed, and diluted in saline. Approximately 100 bacteria were spread on the surface of a 0.22-µm filter which in turn was placed on a TG2PP plate seeded (left) or unseeded (right) with 10^9^*ΔphoA*::Cm cells. In addition, 100 *Δpst*::Km bacteria were spread on top of a 3 filter stack placed on the surface of a TG2PP plate. The plates were incubated at 37 ^∘^C for 48 h

One hundred *Δpst*::Km bacteria (strain BS07) were mixed with increasing numbers (10^5^,10^6^,10^7^,10^8^, or 10^9^) of MG1655 cells and plated on TG2PP. The emergence of PCMs was examined after 48 h (Fig. 2a). When 0 or 10^5^ wild-type bacteria were plated together with the *Δpst*::Km cells, virtually all *Δpst* colonies were recovered. However, higher concentrations of wild-type cells progressively inhibited the emergence of *Δpst* colonies. The inhibition levels were on average 47%, 86%, 99%, and 100% for mixtures containing 10^6^,10^7^,10^8^, and 10^9^ wild-type cells, respectively. This result indicates that as of a certain density the neighboring wild-type cells inhibited the growth of the PCMs. To test whether this growth inhibition by wild-type cells is specific to *pst* mutants, 100 cells of a PHO-constitutive *phoR* mutant (strain RI65) were mixed with 10^9^ wild-type bacteria and plated on TG2PP medium. The inset in Fig. 2a shows that the *phoR* mutants were likewise completely inhibited by the presence of the wild-type cells.

### Mutant frequency and mutation rate of PCMs

The growth inhibition of PCM colonies by wild-type cells may mask the actual frequency of these mutants on the selective plate and disguise their mutation rate. To bypass this inhibition and to assess the real rate of PHO-constitutive mutations, a Luria–Delbrück fluctuation test with a low concentration of bacteria (to circumvent PCM growth inhibition by wild-type bacteria) was conducted. Approximately 1000 MG1655 cells were inoculated in each of 60 wells of a 96-well plate, each containing 0.1 ml TGP medium supplemented with 110 nM glucose and grown for 30 h. This low glucose concentration limits cell yield to ∼5·10^4^ bacteria/ml, which is below the threshold that inhibits the propagation of PCM cells (Fig. 2a). The entire volume of each culture was each plated on a TG2PP plate, and the number of colonies formed at 48 h was counted (Table 1). Only 6 out of 60 plates showed at least one PCM colony. The mutation rate of PCM, calculated by the Luria-Delbrück P_0_ method [36, 37], was 7.9×10^−6^ mutations/cell/generation. The same experiment was repeated using the mutator strain *ΔmutS*::Cm (strain RI1103) in which case 57/60 plates displayed PCM colonies. The high number of mutants allowed us to calculate the mutation rate by additional estimators that rely on medians, such as the Lea–Coulson [38] and Jones method [39]. The calculated mutation rate in this strain was between 9.2×10^−5^ and 1.0×10^−4^ mutations/cell/generation, which is roughly 12 times higher than the mutation rate of the wild-type strain. Thus, the expected number of PCM colonies from a 10^9^ MG1655 culture should be at least 8000, but less than 100 colonies are usually observed at the end of 14 days (Fig. 1). In the case of the *mutS* strain, an even higher number of mutations (∼96,000) would be expected, but an average of only 300 colonies emerged on the selective plate (Fig. 1d).

**Table 1:** Fluctuation test for PCMs in strains MG1655 and *ΔmutS*

	MG1655	*ΔmutS*
Plates with 0 mutants	54	3
Total number of cultures	60	60
*μ*(/cell/generation) (P_0_)^1^	7.9×10^−6^	9.2×10^−5^
*μ*(/cell/generation) (Lea–Coulson)^2^	–	1.01×10^−4^
*μ*(/cell/generation) (Jones)^3^	–	9.2×10^−5^

^1^Luria–Delbrück P_0_ method [36]

^2^Lea and Coulson method [38]

^3^Jones estimator [39]

In addition to the fluctuation test, an alternative strategy was employed to estimate the actual number of plated PCM cells (mutant frequency) on the selective plate (Additional file 1: Figure S2 displays a scheme representing the experimental design). Overnight cultures each containing 10^9^ wild-type MG1655 cells were mixed with increasing numbers of *Δpst*::Cm cells (from 10^3^ to 10^5^) and plated on TG2PP (Table 2, 1st column). After 7 days of incubation, the PCM colonies from each mix were replica plated on L-agar containing chloramphenicol to determine the number of *Δpst*::Cm mutants (3rd column). The 4th column shows the number of spontaneous Cm^S^ PCM mutants that emerged on the plates. Once the number of *Δpst*::Cm cells added is known, their recovery level reveals the extent of their inhibition (5th column). The 6th column shows the expected number of spontaneous PCM cells that were plated on the selective plates by multiplying the number of spontaneous PCM colonies (4th column) by their respective inhibition factor (5th column). For instance, of the 345 colonies that emerged on the 5 plates seeded with the mix of 10^9^ wild-type bacteria + 5000 *Δpst*::Cm cells, only 26 proved to be *Δpst*::Cm, resulting in an inhibition factor of $ \frac {25000}{26}=961 $ fold. Given that on the 5 plates there were in total only 319 spontaneous PCM, the real number of PCMs on these plates should have been 319×961=3.06×10^5^ per 5×10^9^ cells (6.1×10^4^/10^9^ cells). The bottom line in Table 2 shows that the mean inhibition factor was 897 and, therefore, the expected number of PCMs on a plate seeded with a culture of 10^9^ MG1655 cells was 50,000 colonies. The average expected frequency of PCMs in a TG2PP plate seeded with 10^9^ cells is thus 5.0·10^−5^, which is about 6.8 times above the estimated mutation rate of 7.9×10^−6^ obtained by the fluctuation test (Table 1). The ratio [mutant frequency]/[mutation rate] found here is similar to that observed in other systems [36, 40, 41].

**Table 2:** Inferring the frequency of spontaneous PCMs in a mixed culture

5 plates each containing 10^9^	Total PCMs	*Δpst*::Cm	Spontaneous	Inhibition factor	Expected no. of
wt cells + the following no.	at day 7	colonies	PCM colonies	$\left (\frac {\Delta pst\textrm {::Cm}\ \text {plated}}{\Delta pst\textrm {::Cm}\ \text {observed}}\right) $	spontaneous PCMs/ 10^9^
of *Δpst*::Cm cells					cells
(5×) 10^3^	267	7	260	714	3.7×10^4^
(5×) 3×10^3^	269	20	249	750	1.9×10^4^
(5×) 4×10^3^	364	18	346	1111	7.7×10^4^
(5×) 5×10^3^	345	26	319	961	6.1×10^4^
(5×) 10^4^	209	31	178	1612	5.7×10^4^
(5×) 2.5×10^4^	254	114	140	658	1.8×10^4^
(5×) 5×10^4^	977	391	586	639	7.5×10^4^
(5×) 10^5^	1098	686	412	729	6.0×10^4^
Means				897	5.0×10^4^

Five cultures each containing 10^9^ wild-type bacteria were mixed with increasing numbers of *Δpst*::Cm mutants and immediately plated on TG2PP. After 7 days of incubation, the total number of colonies was counted and tested for chloramphenicol resistance. The ratio [ *Δpst* plated]/[ *Δpst* colonies observed] gives the inhibition factor of *Δpst*. The expected no. of PCM/ 10^9^ cells was calculated by multiplying the number of spontaneous PCMs on the 5 plates by the inhibition factor divided by 5. The values in columns 2, 3, and 4 correspond to the sum of mutants found in the five plates

### Mechanism of PCM growth inhibition

In quorum sensing, bacteria send signals to their counterparts or other organisms through the secretion of small molecular weight molecules. In some cases, quorum sensing molecules signalize growth inhibition or cell death [42]. To test whether growth inhibition of PCMs is mediated by a secreted molecule, the filtered spent medium of an MG1655 culture grown overnight in TGP and further incubated in liquid TG2PP for 48 h (to mimic the conditions of PCM selection) was used to resuspend 100 *Δpst* bacteria, which were then plated on TG2PP and incubated for 48 h. Figure 2b shows that the supernatant of the wild-type strain did not inhibit the growth of the *Δpst* colonies. We also tested whether the wild-type cells must be alive in order to inhibit the growth of PCM. 10^9^ wild-type bacteria killed either by immersion in liquid nitrogen or by UV irradiation were mixed with 100 *Δpst* mutants and plated on TG2PP for 48 h. Figure 2c shows that wild-type bacteria killed by either method failed to inhibit the emergence of PCM colonies on the selective plate indicating that the inhibitor cells must be alive to inhibit PCM growth.

**Fig. 3.**
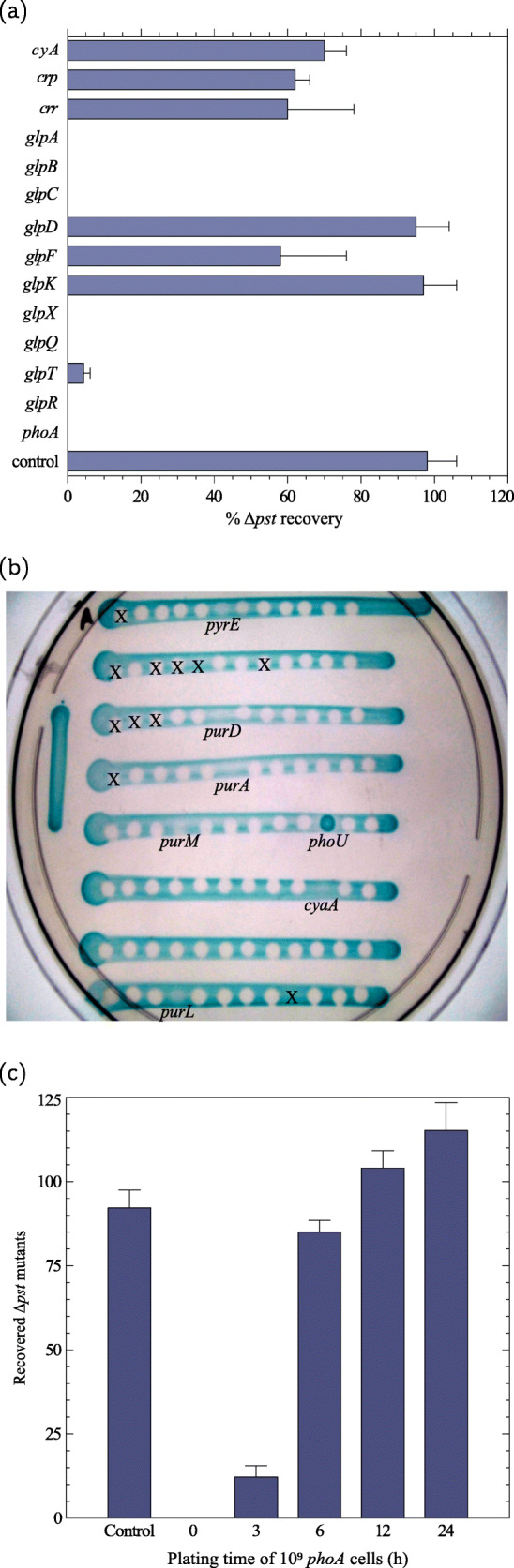
**a** Inhibition of *Δpst* colony growth by single gene knockouts from Keio collection (BW25113 background). One hundred *Δpst* cells were mixed with 10^9^ bacteria carrying individual deletions in each of the following genes: *cyaA*, *crp*, *crr*, *glpA*, *glpB*, *glpC*, *glpD*, *glpF*, *glpK*, *glpX*, *glpQ*, *glpT*, and *glpR*. The plates were incubated for 2–3 days at which time the PCM colonies were counted. “Control” represents the *Δpst* strain plated in the absence of other bacteria and *phoA* serves as a positive control. Each bar represents the mean ± S.E.M. of at least 3 independent cultures. **b** Screening the *E. coli* collection of knockouts for *Δpst* growth inhibition. *Δpst* cells and the library knockouts (Keio collection plate no. 53) were grown overnight in TGP medium. The *Δpst* culture was diluted a hundredfold and 30 *μ* l of this dilution was used to create a linear patch on a TG2PP plate supplemented with XP. Once the *Δpst* patch was dry, 2 *μ* l of each knockout strain was dropped over the patches. The plates were incubated for 48 h at 37 ^∘^C. In the vast majority of cases, a halo was formed inside the patch where the knockout strain was applied, indicating that *Δpst* growth was inhibited by this particular strain. Only a few knockouts described in the main text allowed the growth of *Δpst*, characterized by a bluish color inside the drop. These strains were further tested in a conventional inhibition assay (as in Fig. 3a) to confirm this phenotype. The strong blue color inside the *phoU* drop is because this mutant is a PCM that grows on TG2PP. Of those that did not inhibit *Δpst*, the majority was formed by auxotrophic strains. For instance, in plate 53, the knockouts of *pyrE*, *purA*, *purD*, *purL*, and *purM* did not inhibit *Δpst* growth, but they do not grow or grow very poorly in minimal medium. Halos marked with an X correspond to bacteria that are not part of the Keio collection (see the Keio collection documentation at https://shigen.nig.ac.jp/ecoli/strain/resource/keioCollection/ about). **c** Formation of PCM colonies from apparent cell clusters in the presence of 10^9^ inhibitors. Approximately 100 *Δpst* cells were plated on TG2PP and immediately incubated at 37 ^∘^C. Top-agar carrying 10^9^*phoA* cells was poured over the *Δpst* bacteria at time 0, 3, 6, 12, or 24 h following *Δpst* plating. The plates were then further incubated, such that the total incubation time for each plate was 48 h. The CFU number was counted at the end of the 48-h incubation. The control plates did not contain phoA cells. Each bar represents the mean ± S.E.M. of 5 independent cultures

Other instances of growth inhibition by neighboring cells that demand either cell contact [43–45] or proximity [46] have been reported. To test whether PCM growth inhibition requires the contact between mutants and wild-type bacteria, 100 *Δpst* cells were spread on top of a 0.22-µm membrane placed over a TG2PP plate previously seeded with 10^9^*ΔphoA* cells and incubated for 48 h (Fig. 2d). The negative control consisted of 100 *Δpst* cells placed on a filter on top of an unseeded TG2PP plate. In the control plate, all 100 *Δpst* cells formed blue colonies within 48 h. However, when the filter containing 100 *Δpst* bacteria was placed on top of a TG2PP plate previously seeded with *ΔphoA* cells, not a single colony emerged, even when the bacteria were placed on top of three filters. Thus, growth inhibition does not require contact between the PCMs and the surrounding bacteria. It should be noticed that the *phoA* mutant inhibits PCM growth as does the wild-type strain (see Fig. 3a and c), but unlike the wild-type strain, it cannot generate PCM colonies even after long periods of incubation in the presence of G2P due to its inability to synthesize AP [14].

The selection of PCMs is based on the constitutive expression of AP, which hydrolyzes G2P releasing glycerol in sufficient amounts to allow bacterial growth at a reasonable rate. However, free glycerol may diffuse out of the periplasm of PCM cells and be captured by the numerous wild-type bacteria surrounding them, reducing glycerol concentration available for the growth of the PCMs. If this sequence of events is correct, knockouts of genes related to glycerol metabolism in the inhibitor strain would diminish or abolish the growth inhibition of PCMs. To test this hypothesis, growth inhibition assays were performed with null mutants in genes associated with glycerol uptake and metabolism. The following gene deletions from the Keio collection were used in inhibition assays of PCM cells: *cyaA*, *crp*, *crr*, *glpA*, *glpB*, *glpC*, *glpD*, *glpF*, *glpK*, *glpQ*, *glpR*, *glpT*, and *glpX*. *cyaA* and *crp* encode the CRP-cAMP transcriptional regulator and the bacterial adenylate cyclase, respectively. *crr* codes for the enzyme IIA^Glc^, which, among other things, activates adenylate cyclase [47–49]. *glpA, glpB*, and *glpC* form an operon that encode a glycerol-3-phosphate dehydrogenase complex that converts glycerol-3-phosphate to dihydroxyacetone phosphate under anaerobic conditions [50], and *glpD* codes for an aerobic glycerol-3-phosphate dehydrogenase [51]. The *glpFKX* operon encodes the glycerol facilitator GlpF and the glycerol kinase GlpK, both directly involved in the uptake of glycerol [52], while GlpX is a fructose 1,6-bisphosphatase [53]. GlpR is the repressor of the glycerol-3-phosphate regulon. Noteworthy, some variants of strain MG1655, including the one originally sequenced [54] carry a frameshift deletion in *glpR*, though our MG1655 variant harbors a wild-type *glpR* gene [55]. Finally, *glpQ* and *glpT* code for proteins involved in glycerol-3-phosphate uptake and metabolism [56]. 10^9^ bacteria each carrying single deletions of these genes were each mixed with 100 BW25113 *Δpst* bacteria and plated on TG2PP plates (BW25113 is the parental strain of the Keio knockouts). The growth of *Δpst* colonies was followed for 48 h. Figure 3a shows that the *glpK*, *glpD*, *crp*, *crr*, *cyaA*, and *glpF* knockouts allowed the recovery of more than 50% of the *Δpst* colonies, while the *glpT* deletion inhibited the growth of most, but not all of the colonies. The other knockouts (*glpA*, *glpB*, *glpC*, *glpX*, *glpQ*, and *glpR*) inhibited the growth of all *Δpst* cells.

With the exception of *glpT*, a common characteristic of the knockout mutants that did not completely inhibited *Δpst* growth is that none of them is able to grow with glycerol as the sole C source [52]. Herein, it can be concluded that bacteria that are unable to take up or metabolize glycerol do not inhibit the growth of PCMs. This by itself suggests that the inhibition of PCMs by wild-type cells occurs because most glycerol produced in the periplasm of the PCM cells diffuses out and is consumed by the overwhelming excess of neighboring wild-type bacteria. In fact, the average frequency of PCMs is 5.0·10^−6^ (see Table 2), i.e., for each PCM cell, there are ∼ 20,000 competitor wild-type bacteria on the selective plate seeded with a 10^9^ culture.

To test whether other individual genes are also associated with PCM growth inhibition, the entire Keio library of mutants was screened. Figure 3b shows a picture of a typical experiment in which the mutants of Keio’s plate no. 53 were screened for their effect on *Δpst* growth. In addition to the aforementioned genes, the only other knockout that is not auxotrophic and that did not inhibit the growth of *Δpst* was *clpP* (not shown). ClpP is a protease that together with ClpX and ClpA is associated with protein turnover [57]. The reason why the *clpP* knockout does not inhibit PCM growth is unclear. Finally, to verify that these results are not strain-specific, all gene deletions that did not inhibit BW25113 *Δpst* growth were transferred to strain MG1655 and tested again for growth inhibition of MG1655 *Δpst* (Additional file 1: Figure S4).

### Fitness of PCMs

The present findings have led to the hypothesis that the growth of most PCMs is inhibited by the overwhelming surrounding wild-type cells due to a competition for limited glycerol produced in the PCM periplasms. In addition, it is also possible that the PCMs are less fit than the wild-type strain for growth with glycerol even if both strains are present in equal numbers. To test this assumption, we measured the fitness of the *Δpst* mutant in the presence of the wild-type strain with glycerol as the sole carbon source. One thousand bacteria of each strain—MG1655 and *Δpst* (strain TC02)—were mixed in medium TGlyP (0.2% glycerol as C source) and grown for 72 h with agitation. Samples were withdrawn every 24 h, diluted, and plated on L-agar supplemented with XP to differentiate between the strains. The selection coefficient of strain *Δpst* at the end of 3 days of incubation was −0.67 ± 0.003, an average of three independent experiments. This suggests that in addition to the fact that the PCMs are vastly outnumbered by the wild-type cells on the selective plate, they are also considerably less fit than the wild-type strain (by 67% per generation).

### How do some PCMs manage to grow after all?

Of the ∼ 50,000 PCMs that are expected to be present in a population of 10^9^ bacteria plated on selective TG2PP medium, only a few dozens of PCMs grow and eventually form colonies. However, there is not any impediment, in principle, for the growth of tens of thousands of PCM colonies in 48 h as shown in Additional file 1: Figure S3.

How then do some PCMs manage to escape the inhibition? Is it a stochastic phenomenon or do some lucky mutants have a unique characteristic that permits their growth? We suggest that some PCMs manage to grow and form visible colonies in cases that they form occasional clusters of two or more cells. In these microclusters, the pool of glycerol moieties released by the PCMs is shared among the neighboring cells, which happen to be also PCMs. These sort of mutual feeding allows further replication and the subsequent formation of a PCM colony. To test this hypothesis, one hundred *Δpst* cells were plated on each TG2PP plate. A thin layer of soft-agar containing 10^9^*ΔphoA* cells was added at times 0, 3, 6, 12, and 24 h on top of the plated *Δpst* bacteria, and the plates were further incubated for up to 48 h. Figure 3c shows that when 10^9^*phoA* bacteria were added at time 0 h, no colony was visible after 48 h. However, when the *phoA* cells were added 3 h later, around 12 colonies could be observed, in the 6-h plate and over almost all *Δpst* mutants managed to form colonies. Since the bacteria doubling time on TG2PP is around 2.5 h, the 12 PCM colonies that grew after 3 h could have arisen from clusters of 2 cooperating first-generation siblings. At 6 h, when these clusters consisted of 4–8 siblings, nearly all plated PCMs survived. This suggests that microclusters of PCMs are able to grow and form colonies. Cell microclusters can be formed stochastically upon plating an overnight culture of ∼10^9^ MG1655 wild-type cells containing ∼50,000 PCMs, or from a plated single cell at a late phase of division (see the “Discussion” section).

### A mathematical model for PCM growth and extinction

A mathematical model based on the competition for glycerol produced and released by the PCMs is presented in detail in the Additional file 2. The main hypothesis is that of spatial homogeneity, so that the model can be applied to the entire plate, or to a small portion of it (in this case assuming an absence of any sort of interaction with the surrounding adjacent regions). In addition to spatial homogeneity, the model assumes that both PCMs and wild-type cells equally compete for the glycerol moieties released by the mutant cells. The competition will eventually cause the extinction of both strains following G2P/glycerol depletion, resulting in the tragedy of the commons.

An ideal plate is considered in order to better understand the dynamics of this interaction. The main difference between an ideal plate and the actual TG2PP plate is that in the former the concentration of substrate is constant, while in the actual plate the concentration of G2P decreases with time. Analysis of the model reveals that both strains go to extinction unless they have similar fitnesses. In this case, they can coexist provided there is a minimal initial number of mutants on the plate (see Equation 7 in the Additional file 2). In addition, PCMs may survive alone on the plate if the wild-type strain ability to capture glycerol is very low (see Eq. 14). However, the results of the competition (see the “Fitness of PCMs” section) showed that the wild-type strain is considerably fitter than the PCM in the presence of glycerol as the sole carbon source (Eq. 14 is not satisfied), suggesting that both strains go to extinction in the TG2PP plate. In fact, our data showed that the vast majority of the PCMs would never grow and form colonies and since the wild-type strain cannot grow on G2P both strains would eventually die.

The dynamics trajectory of the competition between wild-type cells and PCMs is detailed in Additional file 2. The model presented can be adjusted to different competition situations (different PCMs to wild-type cell proportions) by adopting different values for *K*,*L*^∗^,*α*,*μ*_*M*_,*μ*_*W*_,*ε*,*δ*,*β*, and *γ*, exposed in Table 2 of the Additional file 2. Let us consider the following values: *K*=10^7^,*L*^∗^=2.1×10^5^,*α*=5,*μ*_*G*_=0.1,*μ*_*M*_=*μ*_*W*_=1,*ε*=*δ*=1, and *β*=*γ*=1 (units are given in Table 2). The initial conditions supplied to the dynamical system (Eq. 3) are *G*(0)=0,*M*(0)=*M*_0_, and *W*(0)=0.1*K*=10^6^, while *M*_0_ is variable. When *M*_0_=1, both populations go to extinction, while for *M*_0_=2, they can coexist. A simulation shows that by applying these *M*_0_ values the minimum PCM to wild-type ratio that allows some level coexistence is 1:10^6^. Indeed, Fig. 2a shows that at a 1:10^6^ proportion, 1% of the PCMs manage to grow and form colonies. As *M*_0_ goes up, increasing thus the PCM to wild-type ratio, the number of PCMs that grow and form colonies also increases (Fig. 2a).

## Discussion

Our results have shown that the classical method for assessing mutant frequency, i.e., colony counting under selective conditions is not always reliable. In the case of PCM selection, it showed an initial rate of less than 2×10^−8^/gene/generation (assuming 100 mutants per 10^9^ bacteria and five *pst* genes as targets), while the actual mutation rate of 1.6×10^−6^/gene/generation turned out to be over hundred-fold higher, and closer to the expected mutation rate in *E. coli* (∼10^−6^/gene/generation [24, 58]).

Krasovec et al. have shown that high cell density partially inhibits the frequency of Rif^R^ mutants on a selective plate via a cell–cell *luxS*-dependent mechanism [10]. According to these authors, lowering bacteria density by 77% increased mutation rate by twofold. PCM mutation rate is also likely to be influenced by this phenomenon, but it only accounts to a small proportion of the nine hundred-fold inhibition observed when 10^9^ bacteria are plated on TG2PP (Table 2). Other examples of bacterial growth inhibition by contact or proximity have been reported [44–46]. In one case, the inhibition was caused by toxins encoded by the CdiAB system from strain EC93, isolated from rat intestines [44]. Another instance was the inhibition caused by strains 25 and 256 isolated from cattle, whose mechanism of inhibition remains unknown [46]. In contrast to the aforementioned systems, stationary phase-dependent contact inhibition reported by Lemonnier et al. [45] bears some similarities with PCM inhibition. In this system, inhibition occurs between isogenic strains, such that the only difference between the inhibitor and inhibited strain is that the former carries a mutation in *glgC* that encodes an enzyme involved in glycogen synthesis. Bacteria carrying a mutation in this gene overproduce glycogen. However, this inhibition system is strain-dependent and was not detected in MG1655, the strain used in our study [45]. In contrast, PCM inhibition was observed in other *E. coli* laboratory strains (Fig. 3) and also in *E. coli* natural isolates (Additional file 1: Figure S1).

Ever since Luria and Delbrück seminal paper [36], it became established that changes in the bacterial genome are not directed to a relevant locus, notwithstanding selective pressures or environmental condition. However, the idea of directed mutagenesis resurfaced when non-lethal selective conditions instead of selection to virus/antibiotic/toxin resistance were employed, a process that has been named “adaptive or directed mutagenesis” [59]. Later, Cairns and others have shown that some mutations arise in response to environmental challenges [3, 59–62]. Adaptive mutations must occur in non-dividing bacteria and only after the bacteria have been in contact with the selective agent and, for that reason, adaptive mutant colonies emerge late on the selective plate. The very existence of adaptive mutations is still disputed, and the controversy remains unresolved [63–67]. Despite the fact that PCM colonies do not emerge before 72 h on the selective plate and that their appearance display a sigmoid curve (features common to adaptive mutations), the high PCM mutation rate, the high frequency of preexisting PCMs, and the extent of their inhibition strongly suggest that most, if not all PHO-constitutive mutations, are preexisting and do not occur after the bacteria have been plated.

It could still be argued that the wild-type bacteria may replicate on the selective plate using traces of carbon contaminants or by using the small amounts of glycerol produced by AP basal level in those strains. Even a few rounds of replication would increase the number of mutant colonies [7, 8]. However, the wild-type strain does not grow at all on the TG2PP plate [14]. The most plausible explanation for the low PCM frequency and late appearance is that the vast majority of the PCMs are inhibited because the overwhelming ancestral neighboring cells seize upon the glycerol moieties produced by the PCMs. Indeed, bacterial strains that are unable to grow on glycerol do not inhibit PCM growth (Fig. 3a).

The production and sharing of public goods is a form of cooperation among organisms that are vulnerable to the exploitation by cheaters. These are individuals in the population that benefit from the public goods without sharing the cost of production. Cheaters are better able to exploit the cooperators in dense populations and when the cheaters themselves are less frequent than the cooperators [68]. However, if the magnitude of cheating increases such that the number of cheaters far exceeds the number of cooperators, the whole community collapses resulting in a situation known as the tragedy of the commons [21, 69, 70]. The unfair competition between cheaters and cooperators is aggravated by the fact that the cooperators, carrying the burden of producing the public goods (AP in our case), display a lower relative fitness compared to the cheaters [71]. Despite the risk of exploitation by cheaters, there are various examples of cooperation mediated by the production of public goods in microorganisms, such as the production and secretion of siderophores that scavenge ferric ions in the extracellular space [72], *β*-lactamase that hydrolyzes *β*-lactam antibiotics [73], or enzymes that facilitate the utilization of sucrose in yeast [74].

The selection and growth inhibition of PCMs might well fit an instance of cheating–cooperation that ultimately results in the collapse of the community. The public good, AP, is produced constitutively by the PCMs and secreted to the periplasm, where it hydrolyzes G2P releasing glycerol that is used as a C source by the entire population. The PCMs, being a minority from the beginning, are overwhelmed by the vast majority of wild-type cells whose AP production is repressed. In the classical tragedy of the commons applied to microbial evolution, the cheaters are usually individuals (mutants) that invade and exploit the population of cooperators for their own benefit [68, 71, 75]. As their fraction in the population increases, the production of public goods dwindles resulting ultimately in the collapse of the entire population [70]. In contrast, here, the cooperators (PCMs) are in the minority and are thus being exploited by the overwhelming majority of cheaters (wild-type bacteria) from the beginning to the point that most PCMs are unable to grow. This situation bears similarities with another instance of bacterial cooperation which is the production of *β*-lactamase that hydrolyzes *β*-lactam antibiotics in the vicinity of the growing colony [73]. Similarly to AP, *β*-lactamase is located in the periplasm and the cooperator’s (*β*-lactamase producers) action against *β*-lactams benefits the neighboring cells, as evidenced by the satellite colonies of susceptible bacteria (cheaters) growing around a single *β*-lactamase producing colony [76]. However, in this case, the growth of the cooperators is not completely hindered by the cheaters and the population does not collapse. The basic difference between cooperation through *β*-lactamase and AP secretion is that the product of the cooperation, glycerol, is almost entirely leached by the overwhelming cheaters while the product of *β*-lactamase activity is the elimination of *β*-lactams in the immediate vicinity allowing first and foremost the growth of its producer and only marginally the growth of neighboring susceptible cells. In addition, coexistence is constrained by the initial dose of the antibiotic, as doses greater than the “minimum inhibitory concentration” preclude the coexistence between resistant and susceptible bacteria [76, 77].

Many bacterial species secrete AP, either to the periplasm or to the extracellular space [78]. Thus, other species besides *E. coli* are also likely to experience cheating from selfish individuals in the population when growing with G2P. It should be noticed that AP secretion to the periplasm is, for the purpose of glycerol release, indistinguishable from secretion to the extracellular space, as the outer membrane allows free passage of small substrates through porin channels [79].

**Fig. 4.**
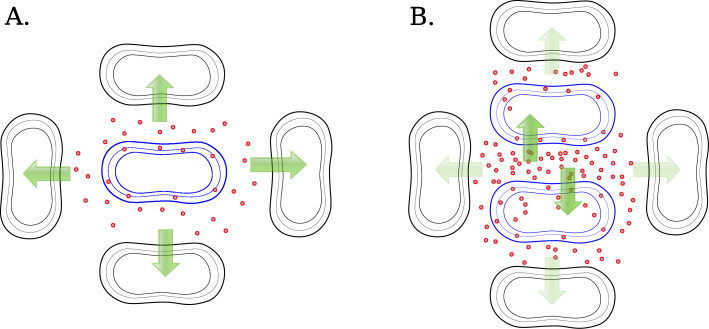
**a** A single PCM (blue bacterium) produces glycerol molecules that diffuse out and are captured by the surrounding wild-type cells. The concentration of glycerol in the PCM vicinity is insufficient to enable the growth of the mutant or the growth of the neighboring wild-type cells, leading to the collapse of the population. , glycerol molecules produced by the PCM. **b** A microcluster of two PCMs mutually feeding each other. Under these conditions, the concentration of glycerol available to the PCMs increases. Glycerol freely moves between PCM cells, being thus sufficient to foment the growth of a colony

The question of how some PCMs do manage to grow and form colonies could best be answered by assuming that the lucky colonies have derived from small clusters of PCM cells. These clusters could be composed of different or identical PCMs. In one rare case, two or more PCM cells would randomly repose close to each other upon plating, and in a second case, the cluster could be composed of a bacterium completing the replication process on the plate resulting in two identical siblings. The evidence presented in Fig. 3c conforms the formation of homogeneous colonies, but it does not rule out the possibility that heterogeneous colonies are formed by different PCM cells. Moreover, these two alternatives are not mutually exclusive. In fact, a back of the envelope calculation shows that the probability that a pair of 2 different PCM cells would repose next to each other on the selective plate is about $ p = 8\times \frac {50,000}{10^{9}} = 0.00040 $, where 8 is an approximation for the number of immediate neighbor cells of each bacterium and 50,000 is the estimated frequency of PCMs in a population of 10^9^ bacteria (based on Table 2). Thus, in a population of 50,000 PCMs, 20 PCM adjacent pairs are expected and a negligible number of 3-cell clusters. This number is in the range of those observed on the selective plates (see Fig. 1). In addition, the general model described in the Additional file 2 (section 1.2) entertains a situation in which the flow of glycerol towards the majority of wild-type cells is restricted by an increasing number of PCMs, simulating thus the formation of clusters. In this particular case, only the mutant cells are able to grow. As mentioned above, PCM clusters could also be homogeneous, i.e., composed of 2 siblings of a PCM cell that landed on the G2PP plate at some advanced step of division. All that is needed is that two or more adjacent PCM cells would be mutually protected from loosing glycerol to the neighboring wild-type cells, allowing them to start replicating and eventually forming a colony. Figure 4 shows a schematic representation of the model depicting the availability of glycerol in a single PCM and in a small cluster of PCMs growing on G2P.

Interestingly, it has been shown that individual cells of *Saccharomyces cerevisae* are unable to utilize sucrose, unless they form microclusters that secrete invertase, an enzyme that hydrolyzes sucrose into glucose and fructose that, unlike sucrose, can be imported by the yeast cells [74]. Thus, the cooperation of *S. cerevisae* cells in the microclusters is the main feature that enables their growth when sucrose is scarce. It has also been shown that similarly to the microclusters of PCMs, the *S. cerevisae* clumps have an advantage over cheaters that do not produce invertase.

## Conclusions

The selection of PCMs on G2P exemplifies an instance in which a high mutant frequency is being masked by the competition between mutants and their wild-type ancestors. This is also a case where cooperators (PCMs) are strongly inhibited by the overwhelming cheaters (wild-type cells), leading to a situation in which neither the cheaters nor the majority of cooperators can grow, resulting in the collapse of the population—a tragedy of the commons. Yet, rare clustering of mutants allows them to grow and form colonies.

## Methods

### Strains, plasmids, and growth media

The bacterial strains used in this study are listed in Additional file 1: Table S1. Unless otherwise stated, bacteria were incubated at 37 ^∘^C under aerobic conditions. LB/L-agar is the standard rich medium [80]. The minimal media TGP and TG2PP were composed of T-salts [15] (0.12 M Tris-HCl, 80 mM NaCl, 20 mM KCl, 20 mM NH_4_Cl, 0.98 mM MgCl_2_.6H_2_O, 2.46 mM Na_2_SO_4_, 2 mM CaCl_2_, 2 µM FeCl_3_, 2 µM ZnCl_2_, pH 7.5) supplemented with 1 mM of the KH_2_PO_4_ and either 0.2% glucose (TGP), 0.2% glycerol-2-phosphate (TG2PP) or 0.2% glycerol (TGlyP).

### Gene knockouts

Genes and operons were deleted using the *λ*-*red* recombinase system as originally described [81, 82]. Briefly, chloramphenicol or kanamycin resistance genes were amplified using plasmid pKD3 or pKD4 as templates and the hybrid primers described below. The amplicons containing the *cat* gene or *kan* genes and 40 bases flanking sequences corresponding to genes *mutS* gene (primers mutS_mut_Fow – ATCACACCCCATTTAATATCAGGGAACCGGACATAACCCCGTGTAGGCTGGAGCTGCTTC and mutS_mut_Rev – GTTAATATTCCCGATAGCAAAAGACTATCGGGAATTGTTACATATGAATATCCTCCTTAG) and the operon *pstSCAB-phoU* (primers pst_m_Fow – GTCTGGTGAATTATTTGTCGCTATCTTTCCCCGCCAGCAGTGTGTAGGCTGGAGCTGCTTC and pst_m_rev – AGGAGACATTATGAAAGTTATGCGTACCACCGTCGCAACTCATATGAATATCCTCCTTAG) were electrotransformed into exponentially growing cultures of strains KM32 or KM44 grown in LB medium supplemented with 1 mM IPTG. Recombinants were selected on L-agar plates containing appropriate antibiotics. Knockout of *mutS* was confirmed by PCR with the primers mutS_ver_Fow and mutS_ver_Rev, and the deletion of *pstSCAB-phoU* was confirmed by PCR and AP assay. When required, gene deletions were transferred to strain MG1655 by P1 transduction.

### P1 transduction

Chromosomal markers were transferred between strains using P1 transduction as described in Miller [80]. Mutants carrying specific deletions were selected on L-agar plates supplemented with the appropriate antibiotic. Further confirmation of the genetic marker was performed by PCR. Following transduction of gene deletions, the antibiotic marker was removed in some instances using plasmid pFLP2 as described [81].

### Selection of PCMs on TG2PP medium

PCMs were selected as described [13, 14]. Briefly, bacteria were grown overnight in TGP medium, washed three times with 0.9% NaCl, and plated on TG2PP supplemented with XP (40 µg/ml). Approximately 10^9^ were plated on each plate, incubated for different time lengths, and counted daily, as specified in the text. Blue colonies were considered PCMs.

### Assessment of Rif^R^ mutant frequency

Bacteria (strain MG1655) grown overnight in medium TGP were washed three times with 0.9% NaCl. Approximately 10^9^ bacteria were plated on TGP plates containing 100 µg/ml rifampicin. Mutant frequency was calculated as the ratio of resistant mutants over the total number of plated bacteria, estimated by CFU counting of bacteria on L-agar plates.

### PCM inhibition assay

*Δpst* cells and the inhibitor strain to be tested were grown overnight in TGP medium. On the next day, both cultures were washed three times with 0.9% NaCl and, unless otherwise noted, mixed as follows: 10^9^ inhibitor cells and 100 *Δpst* bacteria. The bacterial mix was then plated on TG2PP medium. Plates were incubated for 2 days, and the emergence of blue colonies was recorded.

### Survival of wild-type cells on TG2PP plates

Wild-type bacteria were plated on TG2PP as described in the “Selection of PCMs on TG2PP medium” section above. Agar plugs with 5-mm diameter were removed every 24 h from the plate with the help of a glass cannula. Bacteria were eluted from the agar plug by vortexing for 1 min. Bacteria were then diluted and plated on L-agar plates.

### Bacterial lysis by freezing/thawing

Bacteria were grown overnight in TGP medium and concentrated to a final concentration of 10^10^ bacteria/ml. An aliquot of 100 µl was centrifuged, and the bacteria were resuspended in the same volume of lysis solution (1 M Tris pH 8.0, 5 mg/ml lysozyme, 100 mM phenylmethylsulfonyl fluoride, and 1 µl DNase). The suspension was kept on ice for 30 min and then submitted to 6 cycles of freezing/thawing by immersion in liquid nitrogen. The lysed bacteria were centrifuged to precipitate the debris, and the supernatant was kept on ice until further use.

### Fluctuation test

Strains MG1655 and RI103 (MG1655 *ΔmutS*::Cm) were grown overnight in medium TGP. On the next day, the cultures were washed three times in 0.9% NaCl. Approximately one thousand bacteria from each culture were inoculated in each of 60 wells filled with 100 µl TGP medium containing 110 nM glucose and incubated at 37 ^∘^C for 30 h. The total volume of the cultures was then plated on TG2PP plates followed by incubation for 48 h, at which time the frequency of PCMs was assessed. The number of bacteria in each culture (total CFU) was counted in parallel cultures. To calculate the mutation rate of PCMs derived from MG1655, the P_0_ method was employed [36]. The mutation rate of strain RI103 was calculated using the P_0_ method as well as by the Lea and Coulson [38] and Jones [39] methods.

### Competition assay

MG1655 and the *Δpst* mutant (strain TC02) were grown overnight in medium TGP. On the next morning, approximately 1000 cells of each strain were mixed in 5 ml of medium TGlyP and grown at 37 ^∘^C with agitation. Samples were taken at 0, 24, and 48 h, diluted, and plated on L-agar supplemented with XP. The selection coefficient was calculated from the growth rates according to the formula: $ s=\frac {r_{m}-r_{w}}{r_{w}} \ln {2}$, where *r*_*m*_ and *r*_*w*_ represent the growth rate of the mutant and wild-type strain, respectively. The competition was performed in triplicates.

### Growth inhibition of PCM clusters

Overnight cultures of MG1655 *Δpst* (strain TC02) grown in TGP medium were washed and diluted in 0.9% NaCl. Approximately one hundred bacteria were then plated on TG2PP. The plates were incubated at 37 ^∘^C for 0, 3, 6, 12, or 24 h, at which times a layer of soft-agar (6 g/l) containing 10^9^*ΔphoA* cells was poured over the plate. The plates were returned to the incubator until the total time of 48 h, when the number of PCM colonies was counted.

### Screening of the Keio collection

The Keio strains and the *Δpst* mutant (strain TC01) were grown overnight in TGP medium. On the next day, 1 ml of each culture was centrifuged and resuspended in the same volume of 0.9% NaCl. The *Δpst* suspension was diluted 100X and 30 µl portions of this dilution were spotted on a TG2PP plate which upon tilting the plate created linear patches of bacteria. Once the *Δpst* patch was dry, 2 µl of the each Keio strain was dropped over the patch. The plates were incubated for 48 h at 37 ^∘^C, and the growth of *Δpst* inside the circle formed by the Keio strain sample was evaluated. Growth inhibition was characterized by an empty white circle while a circle filled with a blue patch indicated that the Keio strain did not inhibit the growth of *Δpst*. The Keio knockouts that did not inhibit *Δpst* growth were further tested in a conventional inhibition assay to confirm this phenotype.

### PCM growth inhibition through filters

Bacteria (strains TC01(*Δpst*) and RI05 (*ΔphoA*)) were grown overnight in TGP medium. On the next day, cultures were washed three times with 0.9% NaCl and 10^9^ *ΔphoA* cells were spread on a TG2PP plate. A sterile acetate cellulose filter or a stack of 3 filters (pore size 0.22 *μ*m) were placed at the center of the plate and a drop containing approximately 100 *Δpst* cells was spread on the surface of the top filter. Plates were incubated for 48 h and the growth of PCM colonies was evaluated.

## Supplementary Information

**Additional file 1** Table S1 and Figures S1-S4. Table S1. Strains used in this study. Figure S1. Emergence of PHO-constitutive mutants in an *E. coli* natural isolate. Figure S2. Schematic representation of the experiment depicted in Table 2. Figure S3. Standard G2P plate seeded with thousands of PCMs. Figure S4. Absence of inhibition of *Δpst* growth by single gene knockouts (MG1655 background).

**Additional file 2** A mathematical model for PCMs and wild-type bacteria interaction.

## Data Availability

The dataset(s) supporting the conclusions of this article is(are) included within the article (and its additional file(s)).
