# Supplementary material for: Competition for nutritional resources masks the true frequency of bacterial mutants
Source: BMC Biol. 2020 Dec 14;18:194. doi: 10.1186/s12915-020-00913-1 (PMC7737367; doi:10.1186/s12915-020-00913-1)
Supplement: Supplementary file 1 — Additional file 1 Table S1 and Figures S1-S4. Table S1. Strains used in this study. Figure S1. Emergence of PHO-constitutive mutants in an E. coli natural isolate. Figure S2. Schematic representation of the experiment depicted in Table 2. Figure S3. Standard G2P plate seeded with thousands of PCMs. Figure S4. Absence of inhibition of Δpst growth by single gene knockouts (MG1655 background). [file 12915_2020_913_MOESM1_ESM.pdf]

Competition for nutritional resources masks the true frequency of bacterial mutants

Henrique Iglesias Neves, Gabriella Trombini Machado, Taíssa Cristina dos Santos Ramos, Hyun Mo Yang, Ezra Yagil, Beny Spira

Additional File 1

Table S1. Strains used in this study.

Strain	Relevant phenotype or genotype	Source
MG1655	Standard *E. coli* lab strain	(**?**)
BS07	MG1655 Δ*pstSCAB-phoU*:: Km	(**?**)
TC01	MG1655 Δ*pstSCAB-phoU*::Cm	This study
TC02	MG1655 Δ*pstSCAB-phoU*	This study
RI05	MG1655 Δ*phoA*::Km	(**?**)
RI65	MG1655 *pitA phoR*	(**?**)
RI103	MG1655 Δ*mutS*::Cm	This study
RI110	MG1655 Δ*clpP*::Km	Transduction from JW0427
RI111	MG1655 Δ*crp*::Km	Transduction from JW5702
RI112	MG1655 Δ*crr*::Km	Transduction from JW2410
RI113	MG1655 Δ*cyaA*::Km	Transduction from JW3778
RI114	MG1655 Δ*glpA*::Km	Transduction from JW2235
RI115	MG1655 Δ*glpB*::Km	Transduction from JW2236
RI116	MG1655 Δ*glpC*::Km	Transduction from JW2237
RI117	MG1655 Δ*glpD*::Km	Transduction from JW3389
RI118	MG1655 Δ*glpF*::Km	Transduction from JW3898
RI119	MG1655 Δ*glpK*::Km	Transduction from JW3897
RI120	MG1655 Δ*glpR*::Km	Transduction from JW3386
RI121	MG1655 Δ*glpQ*::Km	Transduction from JW2233
RI122	MG1655 Δ*glpT*::Km	Transduction from JW2234
RI123	MG1655 Δ*glpX*::Km	Transduction from JW3896
RI124	MG1655 Δ*phoA*::Km	Transduction from JW0374
BW25113	F^- DE(*araD-araB*)567 *lacZ*4787(del)::*rrnB*-3 LAM- *rph*-1 DE(*rhaD-rhaB*)568 *hsdR*514	Keio collection
RI1494	BW25113 Δ*pstSCAB-phoU*:: Km	Transduction from BS07
JW0427	BW25113 Δ*clpP*::Km	Keio collection
JW5702	BW25113 Δ*crp*::Km	Keio collection
JW2410	BW25113 Δ*crr*::Km	Keio collection
JW3778	BW25113 Δ*cyaA*::Km	Keio collection
JW2235	BW25113 Δ*glpA*::Km	Keio Collection
JW2236	BW25113 Δ*glpB*::Km	Keio Collection
JW2237	BW25113 Δ*glpC*::Km	Keio Collection
JW3389	BW25113 Δ*glpD*::Km	Keio Collection
JW3898	BW25113 Δ*glpF*::Km	Keio collection
JW3897	BW25113 Δ*glpK*::Km	Keio collection
JW3386	BW25113 Δ*glpR*::Km	Keio collection
JW2233	BW25113 Δ*glpQ*::Km	Keio collection
JW2234	BW25113 Δ*glpT*::Km	Keio collection
JW3896	BW25113 Δ*glpX*::Km	Keio collection
JW0374	BW25113 Δ*phoA*::Km	Keio collection
KM32	ΔrecBCD::P*tac-gam-bet-exo* cat	(**?**)
KM44	ΔrecBCD::P*tac-gam-bet-exo* kan	(**?**)

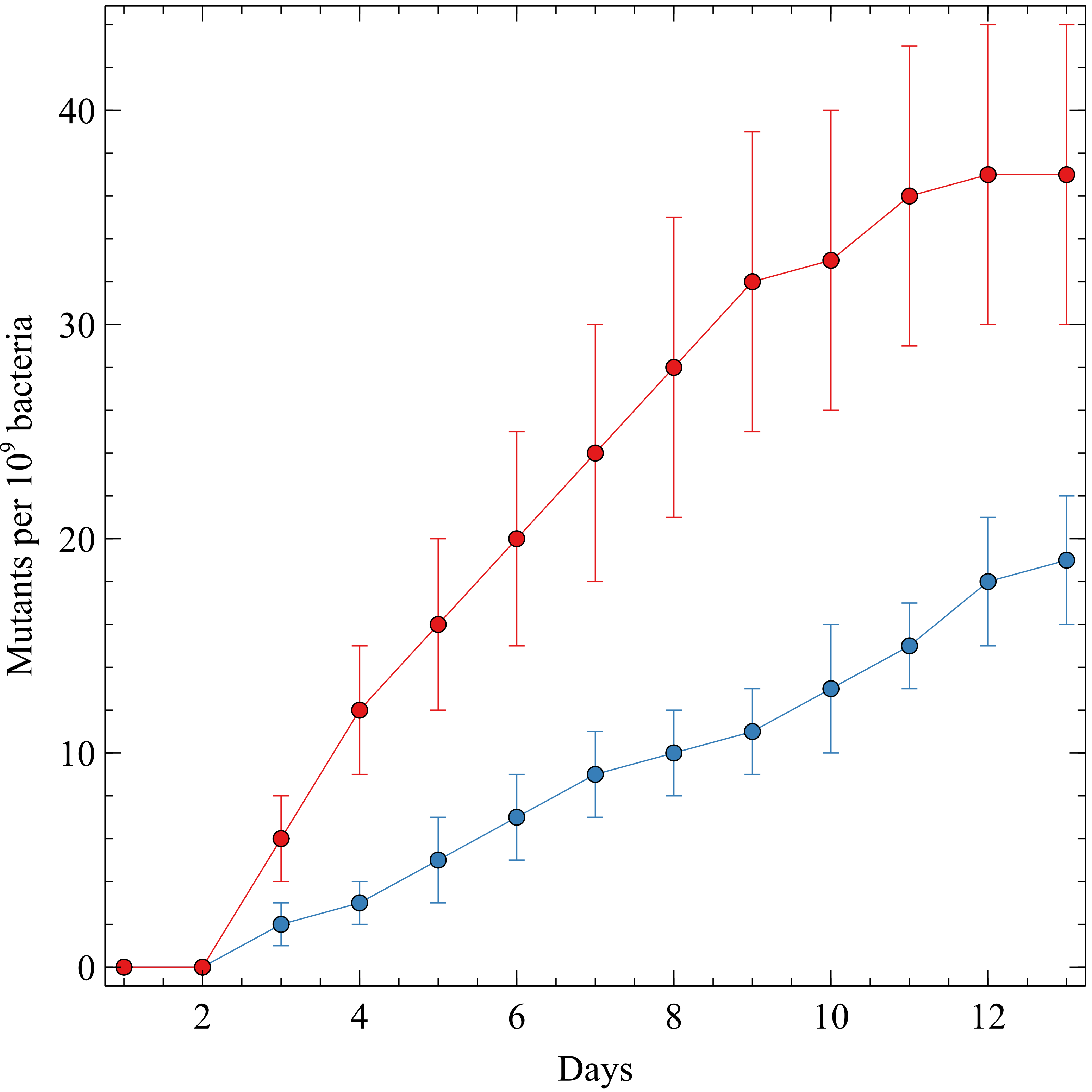

Figure S1. Emergence of PHO-constitutive mutants in strains MG1655 (●) and in *E. coli* (●) natural isolate L25. 10^9 bacteria of each strain were plated on TG2PP and the emergence of PHO-constitutive mutants was followed for 13 days. Each point represents the mean ± S.E.M. of 9 independent cultures.

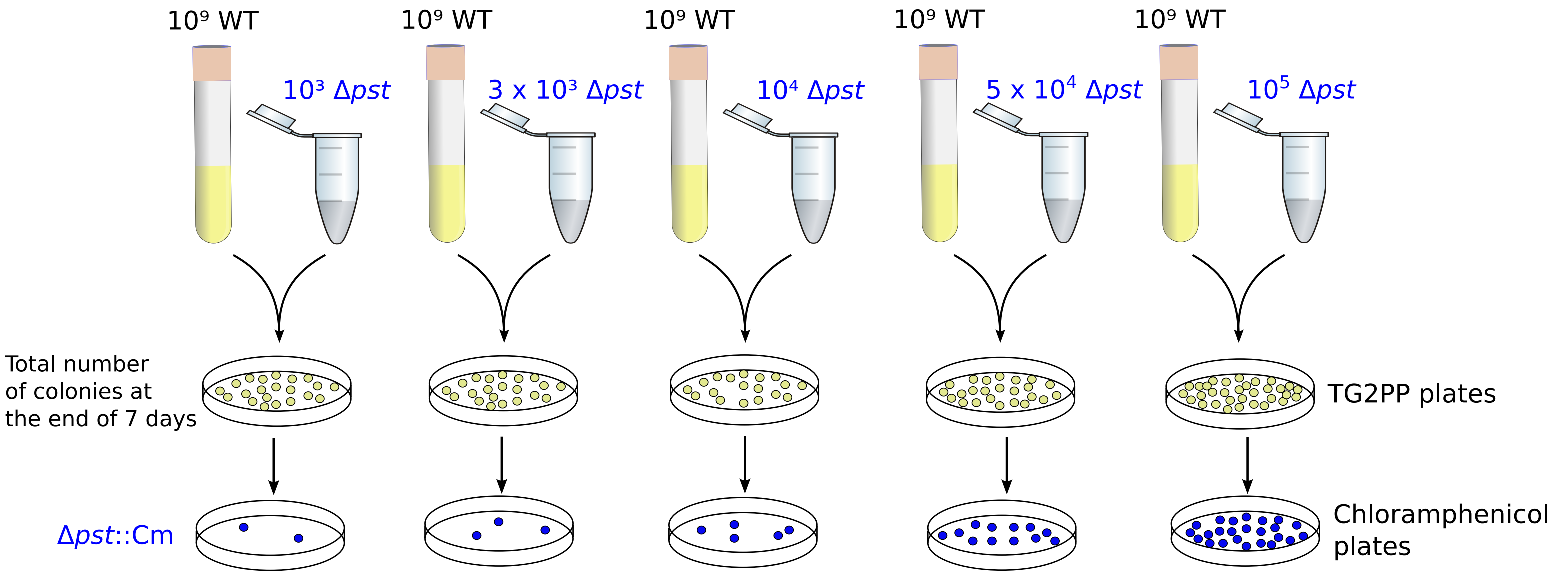

Figure S2. 10^9 wild-type-type cells were mixed with $10^3, 3 \cdot 10^3, 4 \cdot 10^3, 5 \cdot 10^3, 10^4, 2.5 \cdot 10^4, 5 \cdot 10^4$ or 10^5 Δ*pst*::Cm mutants, plated on TG2PP plates and incubated for 7 days at 37°C. For the sake of simplicity only the bacterial mixes containing 1000, 3000, 10,000, 50,000 and 100,000 Δ*pst*::Cm mutants are shown. Forty colonies from each mix were replica plated on L-agar containing chloramphenicol and incubated for 24 h. The proportion of Δ*pst* cells among the total PCMs were used to infer the rate of inhibition of PCM cells growth on TG2PP, as shown in Table 2.

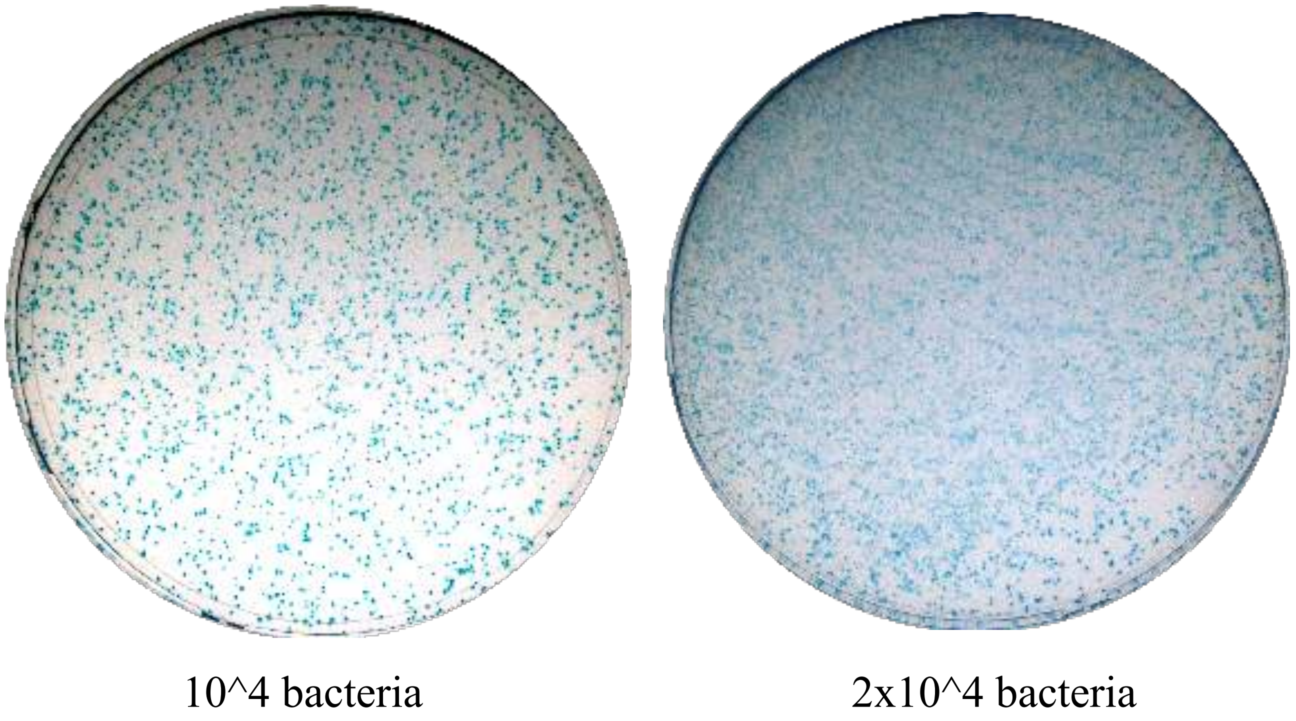

Figure S3. A standard TG2PP plate supports the growth of thousands of small PCM colonies. 10,000 or 20,000 Δ*pst* cells were seeded on TG2PP plates and incubated for 48 h at 37°C.

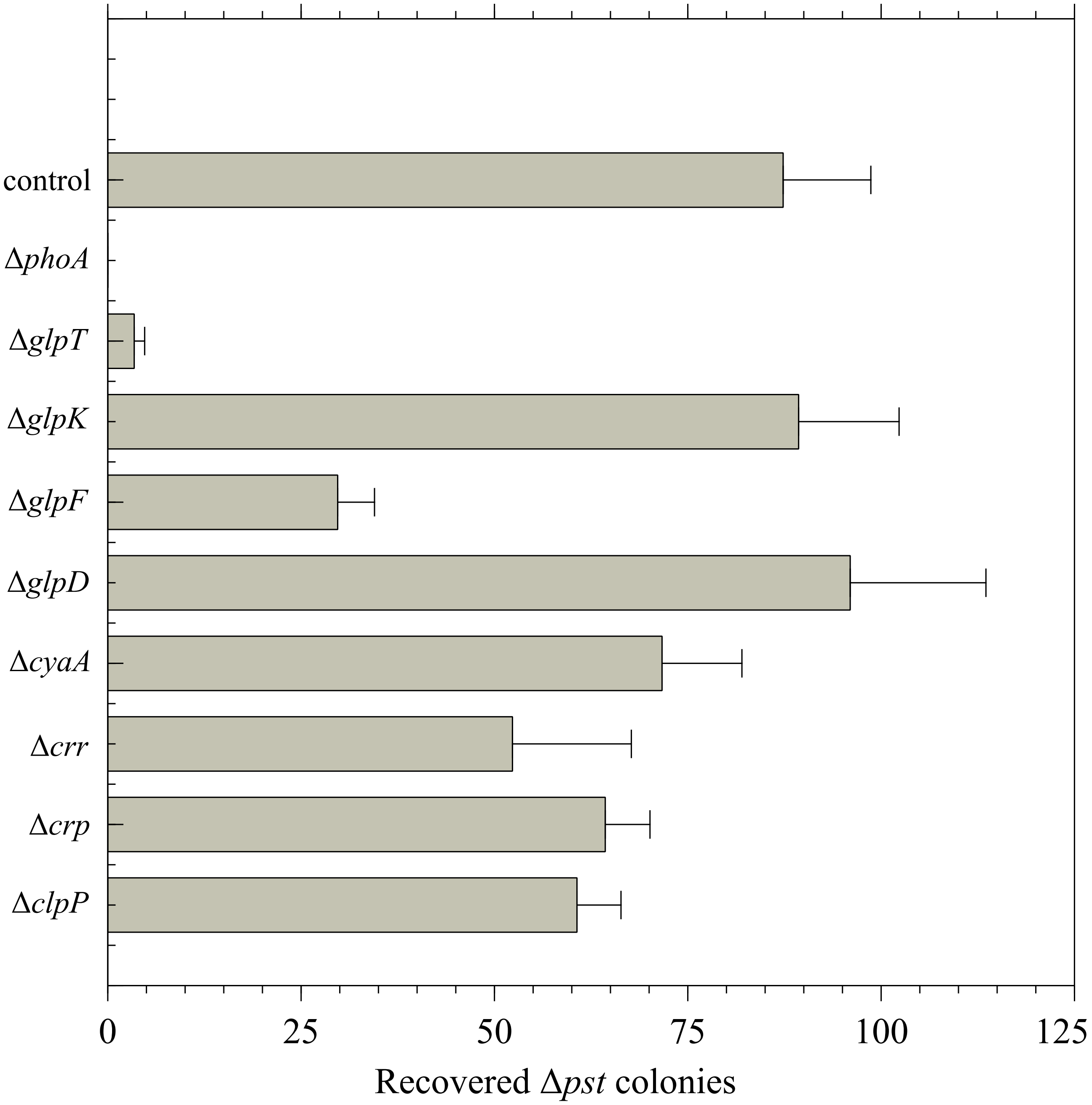

Figure S4. Absence of inhibition of Δ*pst* growth by single gene knockouts (MG1655 background). One hundred Δ*pst* cells were mixed with 10^9 bacteria carrying individual deletions in each of the following genes: *cyaA, crp, crr, glpD, glpF, glpK, glpT* and *phoA* as a positive control. The plates were incubated for 2-3 days at which time the PCM colonies were counted. 'Control' represents the Δ*pst* strain plated in the absence of other bacteria. Each bar represents the mean ± S.E.M. of at least 3 independent cultures.
