# Supplementary material for: Competition for nutritional resources masks the true frequency of bacterial mutants
Source: BMC Biol. 2020 Dec 14;18:194. doi: 10.1186/s12915-020-00913-1 (PMC7737367; doi:10.1186/s12915-020-00913-1)
Supplement: Supplementary file 2 — Additional file 2 A mathematical model for PCMs and wild-type bacteria interaction. [file 12915_2020_913_MOESM2_ESM.pdf]

Competition for nutritional resources masks the true frequency of bacterial mutants

Henrique Iglesias Neves, Gabriella Trombini Machado, Taíssa Cristina dos Santos Ramos, Hyun Mo Yang, Ezra Yagil, Beny Spira

Additional File 2: A mathematical model for PCMs and wild-type bacteria interaction

1. Model formulation

A simple model describing the interaction between the wild-type strain and PCMs on the selective plate was formulated. A pre-determined number of wild-type and mutant strains is spread uniformly on a TG2PP plate, such that there is not further input of any of the two strains. A constant input of G2P is assumed, which is hydrolysed by the PCMs into glycerol and phosphate. All glycerol produced by PCMs is then available to both mutant and wild-type cells, and it is assumed that the phosphate released by G2P hydrolysis does not influence in the dynamics of both strains. In the model, we denote wild-type and mutant populations by W and M, respectively. We consider the production of glycerol only by the mutant strain, which concentration is denoted by G, and assume a fixed input of G2P (the concentration of G2P is denoted by L).

Some glycerol moieties, denoted by G_1, are kept close to their source (special case = glycerol available mostly to the producers), and are consumed only by the mutant cells. However, other glycerol molecules, denoted by G_2, diffuse away from the PCM that produced them (general case = glycerol equally available to all cells), and they can be consumed by both mutant and wild-type cells. We assume that the passage from the special to the general case is described by rate σ. Here, we assume that this rate is positively affected by the number of wild-type cells in the vicinity, and decreases as the number of PCMs increases, that is, $\sigma = \phi G_1 W / (1 + \eta M^{\nu})$, where ϕ is the intrinsic passage rate from the special to the general case, η is a proportionality constant that describes the number of mutant cells required to oppose glycerol drift, and ν describes the intensity of PCMs force in opposing glycerol drift. The two bacterial strains compete for the same resource (glycerol) in a limited space (we denote this limiting space by K, the carrying capacity).

Instead of a TG2PP plate, a general case is considered in the modeling. This model is then applied to the TG2PP plate. A constant influx Ω of G2P is supplied to the environment, and the rate at which glycerol is produced by the mutants is $\alpha_1 LM$, where α is the intrinsic dissociation rate of G2P, and $\alpha_1 L$ is the per-capita production rate of glycerol by the PCMs. The parameter α_2 is the number of glycerol molecules produced by one G2P . The rates at which the mutant and wild-type bacteria take up glycerol are $\beta G_1 M + \beta G_2 M$ (in both cases) and $\gamma G_2 W$ (only in the general case), respectively, where β and γ are the

intrinsic absorption rates of glycerol. A bacterium must absorb more than one glycerol molecule to replicate, hence ε^{-1} and δ^{-1} correspond to the number of glycerol consumed by the PCM and wild-type bacteria, respectively, and their per-capita effective growth rates are $\varepsilon\beta\left(G_1+G_2\right)M\left[1-\left(M+W\right)/K\right]$ and $\delta\gamma G_2 W\left[1-\left(M+W\right)/K\right]$. The per-capita degradation rates of G2P and glycerol are μ_L and μ_G, and the per-capita mortality rates of mutant and wild-type strains are μ_M and μ_W, respectively.

Based on above assumptions and definitions, the model is

$$\begin{cases} \frac{d}{dt}L &= \Omega-\mu_L L-\alpha_1 LM \\ \frac{d}{dt}G_1 &= \alpha_2\alpha_1 LM-\mu_G G_1-\beta G_1 M-\phi G_1\frac{W}{1+\eta M^{\nu}} \\ \frac{d}{dt}G_2 &= \phi G_1\frac{W}{1+\eta M^{\nu}}-\mu_G G_2-\beta G_2 M-\gamma G_2 W \\ \frac{d}{dt}M &= \varepsilon\beta\left(G_1+G_2\right)M\left(1-\frac{M+W}{K}\right)-\mu_M M \\ \frac{d}{dt}W &= \delta\gamma G_2 W\left(1-\frac{M+W}{K}\right)-\mu_W W. \end{cases} \tag{1}$$

To this system of equations, the initial conditions (at $t=0$)

$$\left(L(0)=L_0, G_1(0)=0, G_2(0)=0, M(0)=M_0, W(0)=W_0\right) \tag{2}$$

are given, where L_0, M_0 and W_0 are arbitrarily fixed initial inputs in an environment where glycerol is absent ($G(0)=0$).

The general model described by this system of equations (3) has a very complex passage rate $\sigma=\phi G_1 W/\left(1+\eta M^{\nu}\right)$. For this reason, we deal with a simplified model letting $\sigma\to\infty$, that is, a special case that cannot retain the glycerol molecules. In this case, we have $G_1=0$, $\sigma=\alpha_2\alpha_1 LM$, and changing G_2 by G in the third equation for G_2, we have a simplified model described by

$$\begin{cases} \frac{d}{dt}L &= \Omega-\mu_L L-\alpha_1 LM \\ \frac{d}{dt}G &= \alpha_2\alpha_1 LM-\mu_G G-\beta GM-\gamma GW \\ \frac{d}{dt}M &= \varepsilon\beta GM\left(1-\frac{M+W}{K}\right)-\mu_M M \\ \frac{d}{dt}W &= \delta\gamma GW\left(1-\frac{M+W}{K}\right)-\mu_W W, \end{cases} \tag{3}$$

with the initial conditions

$$(L(0) = L_0, G(0) = 0, M(0) = M_0, W(0) = W_0) \,. \tag{4}$$

Table 1 summarizes the variables described in model (3).

Table 1. Summary of model variables. The dimensions $[G]$ and $[B]$ represent the number of molecules of glycerol and the number of bacteria, respectively.

Symbol	Meaning	Dimension
L	G2P	$[G]$
G	Glycerol	$[G]$
M	Mutant strain of *E. coli*	$[B]$
W	wild-type strain of *E. coli*	$[B]$

Table 2 summarizes the parameters described in model (3).

Table 2. Summary of model parameters and respective mean values. The dimensions $[L]$ and $[t]$ represent the number of molecules of G2P and time ($hour^{-1}$), respectively. The values for glycerol production and its consumption rates are arbitrary and can vary (*).

Symbol	Meaning	dimension	Value
Ω	G2P input rate	$[L]\,[t]^{-1}$	1000
Ω'	G2P input	$[L]$	1000
μ_L	per-capita loss rate of G2P	$[t]^{-1}$	0
μ_G	per-capita loss rate of glycerol	$[t]^{-1}$	0.001
μ_M	per-capita loss rate of mutant strain	$[t]^{-1}$	0.01
μ_W	per-capita loss rate of wild-type strain	$[t]^{-1}$	0.01
α_1	G2P dissociation rate	$[B]^{-1}\,[t]^{-1}$	0.1*
α	glycerol per G2P	$[G]\,[L]^{-1}$	1
β	glycerol absorption by mutant strains rate	$[B]^{-1}\,[t]^{-1}$	0.01*
γ	glycerol absorption by wild-type strains rate	$[B]^{-1}\,[t]^{-1}$	0.01*
ε	glycerol uptaken by mutant strain to grow	$[B]\,[G]^{-1}$	0.01
δ	glycerol uptaken by wild-type strain to grow	$[B]\,[G]^{-1}$	0.01
K	carrying capacity of Petri plate	$[B]$	1000

It is worth stressing the fact that the model given by equation (3) describes an environment supplemented with G2P, which can be dissociated only by PCMs. Nevertheless, this model is easily adapted to an environment containing glycerol by eliminating the first equation (for L), $\alpha_1 L$ is changed to L^*, which now represents a constant influx of glycerol. In addition, $\beta = \gamma$, $\varepsilon = \delta$ and $\mu_M = \mu_W$ (actually, the fitness of the wild-type strain is

higher than that of the PCMs, see section 'Fitness of PCM' in the main text). In other words, both strains are competing only for space, and their coexistence depends only their relative fitnesses.

Let us consider two applications for the simplified model.

(A) An ideal TG2PP plate. Let us assume that the consumed G2P is instantaneously replenished, that is, $\Omega = \mu_L L + \alpha_1 LM$, and, therefore, this substrate is maintained at a constant level (due to $dL/dt = 0$, we have a constant value for L all over time). In this approximation, G2P does not behave as a dynamic variable, and equation (5) becomes

$$\begin{cases} \frac{d}{dt}G &= \alpha L^* M - \mu_G G - \beta GM - \gamma GW \\ \frac{d}{dt}M &= \varepsilon\beta GM\left(1 - \frac{M+W}{K}\right) - \mu_M M \\ \frac{d}{dt}W &= \delta\gamma GW\left(1 - \frac{M+W}{K}\right) - \mu_W W, \end{cases} \tag{5}$$

where $L^* = \alpha_1\Omega'$ represents a constant input of G2P. The reason behind the assumption of a constant input of G2P in an ideal TG2PP plate is to allow the possibility of non-extinction of both *E. coli* strains.

(B) A real TG2PP plate. In this case, an initial amount of G2P is supplied, letting $L^* = 0$ in equation (5), resulting in

$$\begin{cases} \frac{d}{dt}L &= -\mu_L L - \alpha_1 LM \\ \frac{d}{dt}G &= \alpha\alpha_1 LM - \mu_G G - \beta GM - \gamma GW \\ \frac{d}{dt}M &= \varepsilon\beta GM\left(1 - \frac{M+W}{K}\right) - \mu_M M \\ \frac{d}{dt}W &= \delta\gamma GW\left(1 - \frac{M+W}{K}\right) - \mu_W W, \end{cases} \tag{6}$$

with the initial conditions given by equation (4). In a real TG2PP plate, both mutant and wild-type strains go to extinction after the complete consumption of glycerol.

1..1 Analysis of the simplified model

The simplified model describing an ideal TG2PP plate (case A) given by equation (5), is analyzed by determining the equilibrium points (all derivatives are zero) and their stability.

Equation (5) has the equilibrium point $P = (\bar{G}, \bar{M}, \bar{W})$ obtained as the positive solution(s) of a non-linear system of equations given by

$$\begin{cases} \alpha L^* M - \mu_G G - \beta G M - \gamma G W = 0 \\ \left[\varepsilon\beta G\left(1 - \frac{M+W}{K}\right) - \mu_M\right] M = 0 \\ \left[\delta\gamma G\left(1 - \frac{M+W}{K}\right) - \mu_W\right] W = 0. \end{cases} \tag{7}$$

The local stability of the equilibrium point P is determined by the eigen-values of the Jacobian matrix J corresponding to the system of equations (5), which is given by

$$J = \begin{bmatrix} -\beta M - \gamma W - \mu_G & \alpha L^* - \beta G & -\gamma G \\ \varepsilon\beta M\left(1 - \frac{M+W}{K}\right) & \varepsilon\beta G\left(1 - \frac{2M+W}{K}\right) - \mu_M & -\frac{\varepsilon\beta}{K} G M \\ \delta\gamma W\left(1 - \frac{M+W}{K}\right) & -\frac{\delta\gamma}{K} W G & \delta\gamma G\left(1 - \frac{M+2W}{K}\right) - \mu_W \end{bmatrix}, \tag{8}$$

evaluated at the equilibrium point $P = (\bar{G}, \bar{M}, \bar{W})$.

When two strains are competing for the same nutrient produced by only one of them, we have three equilibrium points: extinction (1), coexistence of both strains (2), or survival of the mutant strain only (3).

(1) Extinction of both populations – P^0 From equation (7), it is easy to verify that when $M = 0$ and $W = 0$, we have $G = 0$. This is the trivial (extinction) equilibrium point $P^0 = (0, 0, 0)$. (This is the only equilibrium if $L^* = 0$.)

The Jacobian matrix J given by equation (8) evaluated at the trivial equilibrium point P^0 is

$$J^0 = \begin{bmatrix} -\mu_G & \alpha L^* & 0 \\ 0 & -\mu_M & 0 \\ 0 & 0 & -\mu_W \end{bmatrix},$$

which has corresponding eigen-values $\lambda_1 = -\mu_G$, $\lambda_2 = -\mu_M$ and $\lambda_3 = -\mu_W$. Hence, P^0 is locally asymptotically stable (LAS) [1].

(2) Coexistence of both populations – P^* From equation (7), in order to allow coexistence $M \neq 0$ and $W \neq 0$, the terms in brackets of the last two equations of (7) must be zero simultaneously, which is true if

$$\frac{\delta\gamma}{\mu_W} = \frac{\varepsilon\beta}{\mu_M}. \tag{9}$$

Understanding the quotient as fitness, the coexistence of PCM and wild-type cells is possible if both have similar fitnesses.

For the sake of simplicity, let consider $\delta = \varepsilon$, $\gamma = \beta$ and $\mu_M = \mu_W$. In this case, the last two equations are summed (both equations are the same), resulting in one equation for X, with $X = M + W$, where $M = \nu X$ and $W = (1-\nu)\,X$, and $0 \leqslant \nu \leqslant 1$. Hence, equation (5) becomes

$$\begin{cases} \dfrac{d}{dt}G &= \alpha L^* \nu X - \mu_G G - \beta G X \\ \dfrac{d}{dt}X &= \varepsilon\beta G X \left(1 - \dfrac{X}{K}\right) - \mu_M X \end{cases} \tag{10}$$

Under steady state, $X = 0$ (trivial equilibrium P^0) or X obeys $P_2(X) = 0$, where $P_2(X)$ is a second degree polynomial

$$P_2(X) = \varepsilon\beta\alpha\nu L^* X^2 - \beta K\left(\varepsilon\alpha\nu L^* - \mu_M\right) X + K\mu_M\mu_G, \tag{11}$$

which has 0 or 2 positive roots. There are two conditions that result in positive roots for equation (11):

(a) A weak condition given by

$$\varepsilon\alpha\nu L^* > \mu_M. \tag{12}$$

When $\beta \to \infty$ (perfect fitness, ability of transforming G2P in glycerol) and $K \to \infty$ (unlimited nutritional sources and space), the equation for X in (17) becomes $dX/dt = \varepsilon\alpha\nu L^* X - \mu_M X$, showing that M can survive (or persist) if $\varepsilon\alpha\nu L^* > \mu_M$, in which case $dX/dt > 0$. This weak condition is related to perfect adapted bacteria in a boundless habitat.

(b) The stringent condition is

$$\beta > \beta_0 \equiv \frac{4}{\nu}\frac{\varepsilon\alpha L^*\mu_M\mu_G}{K\left(\varepsilon\alpha L^* - \frac{1}{\nu}\mu_M\right)} \qquad \text{or} \qquad K > K_0 \equiv \frac{4}{\nu}\frac{\varepsilon\alpha L^*\mu_M\mu_G}{\beta\left(\varepsilon\alpha L^* - \frac{1}{\nu}\mu_M\right)^2}. \tag{13}$$

The condition $\beta > \beta_0$ (or $K > K_0$) states that the fitness β (or the carrying capacity K) must be higher than its critical value β_0 (or K_0) in order to mutant strain to surviving; otherwise it goes to extinction.

Hence, when the conditions (12) and (13) are satisfied, two positive equilibrium points of coexistence arise, which are $P_-^* = (\bar{G}_-, \nu\bar{X}_-, (1-\nu)\,\bar{X}_-)$ and $P_+^* = (\bar{G}_+, \nu\bar{X}_+, (1-\nu)\,\bar{X}_+)$, where $\bar{X}_\pm$ are given by

$$\begin{cases} \bar{X}_- &= \dfrac{K\left(\varepsilon\alpha L^* - \frac{1}{\nu}\mu_M\right)}{2\varepsilon\alpha L^*}\left[1 - \sqrt{1 - \dfrac{4}{\nu}\dfrac{\varepsilon\alpha L^*\mu_M\mu_G}{K\beta\left(\varepsilon\alpha L^* - \frac{1}{\nu}\mu_M\right)^2}}\right] \\ \bar{X}_+ &= \dfrac{K\left(\varepsilon\alpha L^* - \frac{1}{\nu}\mu_M\right)}{2\varepsilon\alpha L^*}\left[1 + \sqrt{1 - \dfrac{4}{\nu}\dfrac{\varepsilon\alpha L^*\mu_M\mu_G}{K\beta\left(\varepsilon\alpha L^* - \frac{1}{\nu}\mu_M\right)^2}}\right], \end{cases} \tag{14}$$

and $\bar{G}_\pm$ is obtained from

$$\bar{G}_\pm = \frac{\alpha\nu L^*\bar{X}_\pm}{\mu_G + \beta\bar{X}_\pm}. \tag{15}$$

The Jacobian matrix J given by equation (8) evaluated at the equilibrium point of coexistence P^*, which represents P_-^* and P_+^*, is given by, using equation (17) at steady state,

$$J^* = \begin{bmatrix} -\alpha\nu L^*\dfrac{\bar{X}}{\bar{G}} & \mu_G\dfrac{\bar{G}}{\bar{X}} \\ \mu_M\dfrac{\bar{X}}{\bar{G}} & -\dfrac{\varepsilon\beta}{K}\bar{X}\bar{G} \end{bmatrix}.$$

The corresponding characteristic equation is given by

$$\lambda^2 + \left(\alpha\nu L^*\frac{\bar{X}}{\bar{G}} + \frac{\varepsilon\beta}{K}\bar{X}\bar{G}\right)\lambda + \alpha\nu L^*\frac{\varepsilon\beta}{K}\bar{X}^2 - \mu_M\mu_G = 0,$$

where the independent term of λ is rewritten, using equation (11), as

$$a_0 \equiv \alpha\nu L^*\frac{\varepsilon\beta}{K}\bar{X}^2 - \mu_M\mu_G = \beta K\left(\varepsilon\alpha\nu L^* - \mu_M\right)\bar{X} - 2\mu_M\mu_G,$$

which is zero at

$$\bar{X}_0 = 2\frac{\mu_M \mu_G}{\beta K \left(\varepsilon\alpha\nu L^* - \mu_M\right)},$$

showing that $a_0 < 0$, for $\bar{X} < \bar{X}_0$, and $a_0 > 0$, for $\bar{X} > \bar{X}_0$. By introdusing $\bar{X}_0$ into equation (11), $P_2(\bar{X}_0)$ is given by

$$P_2(\bar{X}_0) = -\mu_M \mu_G K \left(\beta - \beta_0\right),$$

showing that, when $\varepsilon\alpha\nu L^* > \mu_M$ and $\beta > \beta_0$, $\bar{X}_-$ and $\bar{X}_+$ exist, and $\bar{X}_- < \bar{X}_0 < \bar{X}_+$. Hence, when P_*^- and P_*^+ exist, P_*^+ is always LAS, while P_*^- is always unstable.

Hence, the relative position of the given initial conditions (G_0, M_0, W_0) with respect to the coordinates of $P_-^* = (\bar{G}_-, \nu\bar{X}_-, (1-\nu)\,\bar{X}_-)$ divides attracting regions (or basins) of stable $P^0 = (0,0,0)$ and $P_+^* = (\bar{G}_+, \nu\bar{X}_+, (1-\nu)\,\bar{X}_+)$. The curve that separates two attracting basins is called a separatrix. The bifurcation diagram shown in Figure 1 illustrates qualitatively the attraction of stable equilibria P^0 and P_+^* as a function of β (a similar diagram is obtained for the carrying capacity, by substituting β with K and β_0 with K_0) depending on the initial conditions.

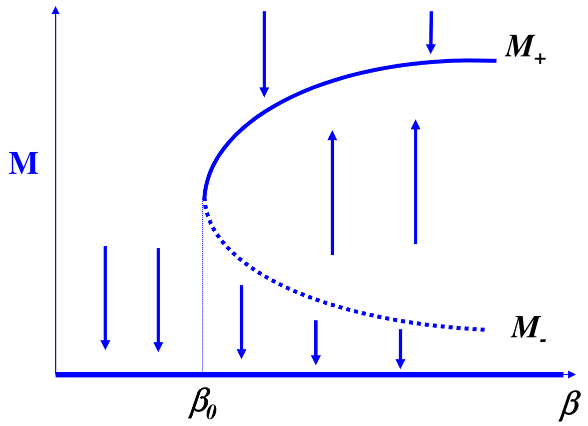

Figure 1. Bifurcation diagram: depending on the relative position of the initial value M_0 with respect to the value M_-, trajectories are towarded to one of the stable equilibria P^0 and P_+^*.

Figure 1 shows that when $\beta < \beta_0$, disregarding the initial conditions (G_0, M_0, W_0), all trajectories approach P^0. However, for $\beta > \beta_0$, all trajectories approach P^0 or P_+^* depending on the initial conditions positioned at P_*^- except $M(0)$, that is, $(G_0 = \bar{G}_-, M_0 = \nu\bar{X}_- + \zeta, W_0 = (1-\nu)$ [illegible] if $\zeta < 0$, the trajectories approach P^0; otherwise if $\zeta > 0$, the trajectories approach P_+^*. In the figure, M_- and M_+ stand for $\bar{M}_- = \nu\bar{X}_-$ and $\bar{M}_+ = \nu\bar{X}_+$, respectively.

When condition (12) or (13) are not satisfied, P_-^* and P_+^* do not exist, and there is not

an analytical expression (coordinates of P^*_-) that divides the attracting regions of extinction (P^0) and coexistence (not P^*_+ anymore). The separatrix that divides the attracting regions is obtained numerically (as well as the coexistence point) and is essentially a quotient between the initial conditions M_0 and W_0, that is, $q = M_0/W_0$, assuming that G_0 is arbitrarily fixed. The dependence with q arises due to the fact that both strains are ruled by the same dynamical equation.

(3) Only the PCM survives – P^M From equation (7), if $M \neq 0$ and $W = 0$, two positive equilibrium points that exclude the wild-type strain arise. One is $P^M_- = (\bar{G}_-, \bar{M}_-, 0)$ and the other is $P^M_+ = (\bar{G}_+, \bar{M}_+, 0)$, where $\bar{M}_\pm$ are given by equation (14), letting $\nu = 1$ (hence, $W = 0$) and $\bar{G}_\pm$ is obtained from equation (15) by changing X with M. The conditions for the existence of P^M_- and P^M_+ are the same those obtained earlier (equations (12) and (13) letting $\nu = 1$).

The Jacobian matrix J given by equation (8) evaluated at the equilibrium point in the absence of the wild-type strain P^M, which represents P^M_- and P^M_+, is given by equation (5) at the steady state,

$$J^*_0 = \begin{bmatrix} -\alpha L^* \dfrac{\bar{M}}{\bar{G}} & \mu_G \dfrac{\bar{G}}{\bar{M}} & -\gamma \bar{G} \\ \mu_M \dfrac{\bar{M}}{\bar{G}} & -\dfrac{\varepsilon\beta}{K}\bar{M}\bar{G} & -\dfrac{\varepsilon\beta}{K}\bar{M}\bar{G} \\ 0 & 0 & \delta\gamma\bar{G}\left(1 - \dfrac{\bar{M}}{K}\right) - \mu_W \end{bmatrix},$$

resulting in the characteristic equation

$$\left[\lambda^2 + \left(\alpha L^* \frac{\bar{M}}{\bar{G}} + \frac{\varepsilon\beta}{K}\bar{M}\bar{G}\right)\lambda + \alpha L^* \frac{\varepsilon\beta}{K}\bar{M}^2 - \mu_M\mu_G\right]\left[\lambda + \mu_W - \delta\gamma\bar{G}\left(1 - \frac{\bar{M}}{K}\right)\right] = 0.$$

The first bracket is a second degree polynomial, that is been analysed by assuming the absence of wild-type cells. When the wild-type population is removed ($W = 0$, letting $\nu = 1$), when $\varepsilon\alpha L^* < \mu_M$ there is only P^0 which is LAS; however when $\varepsilon\alpha L^* > \mu_M$, if $\beta < \beta_0(\nu = 1)$ there is only P^0 which is LAS, while if $\beta > \beta_0(\nu = 1)$ there are three equilibrium points, where two two of them - P^0 and P^M_+ are LAS, while P^M_- is unstable. In this case, P^M_- divides the attracting regions of P^0 and P^M_+. However, in the presence of

a wild-type population there is another condition to be satisfied: the second bracket. In that case, P_+^M is LAS when second bracket has negative eigen-value; on the contrary, P_+^* is not stable any more, and both strains go to extinction (P^0). The second bracket has an eigen-value $\lambda = -(\mu_W - \gamma^c)$, where γ^c is given by

$$\gamma^c = \delta\gamma\bar{G}_+ \left(1 - \bar{M}_+/K\right), \tag{16}$$

which is a threshold value for the replication rate γ. Hence, P_+^M is LAS if $\gamma^c < \mu_W$, that is, the mortality rate is higher than the critical replication rate of the wild-type cells.

It is worth stressing the fact that the condition (16) bears biological meaning. By writing the third equation of (5) around the stable equilibrium value P_+^M, that is, $G = \bar{G}_+$ and $M = \bar{M}_+$, the dynamics (in the presence of the wild-type strain) becomes

$$\frac{d}{dt}W = \left[\delta\gamma\bar{G}_+ \left(1 - \frac{\bar{M}_+}{K}\right) - \mu_W\right] W = -\left(\mu_W - \gamma^c\right) W.$$

This equation near P_+^M states that wild-type population goes to extinction only if its relative fitness is low ($\gamma^c < \mu_W$), then $dW/dt < 0$, the wild-type strain approaches 0 and only the mutant population survives (P_+^M). When the wild-type fitness is higher ($\gamma^c > \mu_W$), $dW/dt > 0$ and the wild-type population grows, competes with the PCMs for nutrients, and, as consequence, both populations go to extinction (P^0).

Summary A model for the nutritional competition between the PCMs and wild-type cells was described by equation (5), in which the medium in the plate contained abundant G2P, but only the mutant strain is capable to hydrolyse G2P into glycerol. Broadly, when $\beta < \beta_0$ (or $K < K_0$), both populations go to extinction ($P^0 = (0, 0, 0)$). Conversely, when $\beta > \beta_0$ (or $K > K_0$), either both populations go to extinction (P^0), or only the PCMs persist ($P_+^M = (\bar{G}_+, \bar{M}_+, 0)$). The separatrix is been given by $P_-^M = (\bar{G}_-, \bar{M}_-, 0)$. However, coexistence of both strains occurs only if $\delta\gamma\mu_M = \varepsilon\beta\mu_W$: either both populations go to extinction (P^0), or both strains persist ($P_+^* = (\bar{G}_+, \bar{M}_+, \bar{W}_+)$). In this case, the separatrix is given by $P_-^* = (\bar{G}_-, \bar{M}_-, \bar{W}_-)$.

To achieve a non-zero solution, a weak condition (12) must be satisfied. This condition

is necessary but not sufficient, due to the fact that neither the bacterial consumption of glycerol (β) nor the limitation of space (K) is taken into account. Hence, a strong condition (13) is required linking the positive solution with the threshold β_0 (or K_0). Notice that as K (or β) increases, β_0 (or K_0) decreases, enabling the survival of the mutant strain or coexistence of both PCM and wild-type strains.

With the aim of simulating a numerical solution (5), values corresponding to the parameters given in Table 2 were proposed. Letting $\nu = 0.02$, the threshold values are $\beta_0 = 8 \times 10^{-6}$ $hours^{-1}$ and $K_0 = 0.8$, and the equilibrium points have coordinates $P_-^* = (100.01, 0.02, 0.098)$ and $P_+^* = (199.96, 9.998, 489.90)$. Figure 2 shows the role of the initial condition in the dynamical system.

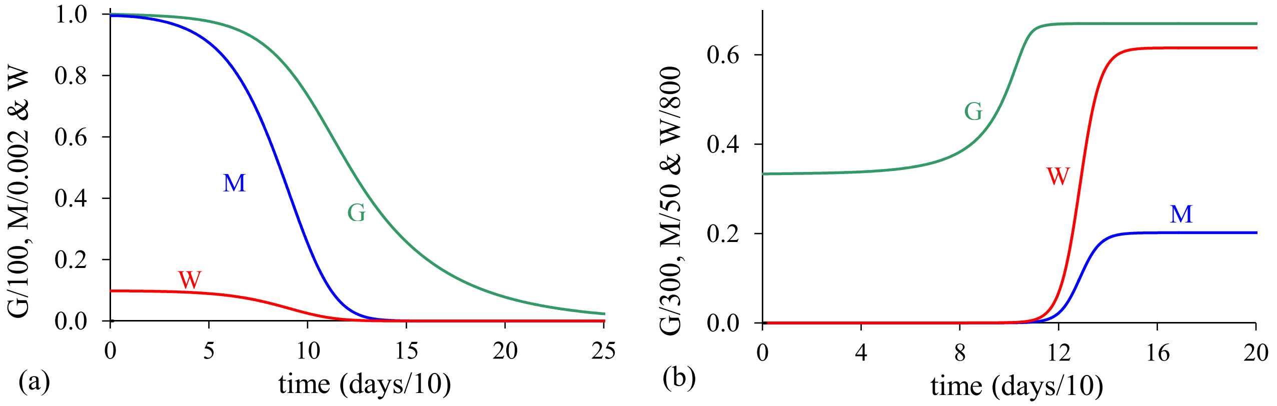

Figure 2. Dynamical trajectories of G, M and W: trajectories achieve equilibrium P^0 (a) or P_+^* (b) depending on the initial conditions with respect to the unstable P_-^*. The actual values of G, M and W are obtained by multiplying the values in the curves by the denominators in the vertical axis.

In Figure 2, we supply as initial condition the values of the coordinates of P_-^* except M (see Figure 1): $G(0) = \bar{G}_-$, $M(0) = \bar{M}_- + \omega$ and $W(0) = \bar{W}_-$. In Figure 2(a), $\omega = -0.00001$ and the extinction of both strains P^0 is the attractor, while in Figure 2(b) $\omega = 0.00001$ and the coexistence of both strains P_+^* is the attractor. The actual values of G, M and W are obtained by multiplying the values in Figure 2 by the denominators in the vertical axis. It is worth noticing that condition (9) must be satisfied in order to obtain coexistence.

Suppose that the initial condition uses the values provided in Table 6. Let us assume that in the plate there is abundant G2P, but not glycerol. Mutant and wild-type strains are added such that the initial conditions are $G(0) = 0$, $M(0) = pK$ and $W(0) = 0.9K$, where p is allowed to vary (notice that $q = M(0)/W(0) = p/0.9$). If $p \leqslant 0.0102$ (or $q \leqslant 1.133\%$), both strains go to extinction and glycerol is not produced any more, but If $p \geqslant 0.0103$

(or $q \geqslant 1.144\%$), both strains coexist and glycerol is produced. The higher the value of p, the higher of coexistence of both strains (because there is a high concentration of glycerol produced by the PCMs).

Finally, from equation (7) or equation (11), it is clearly that when $L^* = 0$, that is, in the absence of a constant input of G2P, both mutant and wild-type strains go to extinction just after the complete depletion of the initial input of G2P. This conclusion is valid for a real TG2PP plate (case B) given by equation (6), i .e., the only equilibrium is the extinction of both population. In this case, after an initial increase of both populations, they go to extinction by approaching the trivial equilibrium $P = (\bar{L} = 0, \bar{G} = 0, \bar{M} = 0, \bar{W} = 0)$.

1..2 Brief notes on the general model

The general model is applied to an ideal TG2PP plate, by letting $\nu = 1$. Assuming that the consumed G2P is instantaneously replenished, that is, $\Omega = \mu_L L + \alpha_1 LM$, and, therefore, this substrate is maintained at a constant level (due to $dL/dt = 0$, we have a constant value for L at all times). In this approximation, G2P does not appear as a dynamic variable, and equation (1) becomes

$$\begin{cases} \dfrac{d}{dt}G_1 &= \alpha L_0 M - \mu_G G_1 - \beta G_1 M - \phi G_1 \dfrac{W}{1+\eta M} \\ \dfrac{d}{dt}G_1 &= \phi G_1 \dfrac{W}{1+\eta M} - \mu_G G - \beta G_2 M - \gamma G_2 W \\ \dfrac{d}{dt}M &= \varepsilon\beta\left(G_1 + G_2\right) M \left(1 - \dfrac{M+W}{K}\right) - \mu_M M \\ \dfrac{d}{dt}W &= \delta\gamma G_2 W \left(1 - \dfrac{M+W}{K}\right) - \mu_W W. \end{cases} \tag{17}$$

where $\alpha = \alpha_2\alpha_1$, and L_0 is a constant input of G2P. The reason behind a constant input of G2P by assuming ideal TG2PP plate is the possibility of non-extinction of the *E. coli* strains. In this model, the passage from the special case to the general one is inversely proportional to the size of the mutant population (see Figure 4 in the main text).

Equation (17) has the equilibrium point $P = (\bar{G}_1, \bar{G}_2, \bar{M}, \bar{W})$ obtained as the positive

solution(s) of a non-linear system of equations given by

$$\begin{cases} \alpha L_0 M - \mu_G G_1 - \beta G_1 M - \phi G_1 \dfrac{W}{1+\eta M} = 0 \\ \phi G_1 \dfrac{W}{1+\eta M} - \mu_G G_2 - \beta G_2 M - \gamma G_2 W \\ \left[\varepsilon\beta \left(G_1 + G_2 \right) \left(1 - \dfrac{M+W}{K} \right) - \mu_M \right] M = 0 \\ \left[\delta\gamma G_2 \left(1 - \dfrac{M+W}{K} \right) - \mu_W \right] W = 0. \end{cases} \tag{18}$$

The local stability of the equilibrium point P is determined by the eigen-values of the Jacobian matrix J corresponding to the system of equations (17), which is given by

$$\mathrm{J} = \begin{bmatrix} -\beta M - \phi\frac{W}{1+\eta M} - \mu_G & 0 & \alpha L_0 - \beta G_1 + J_3 & -\phi\frac{G_1}{1+\eta M} \\ \phi\frac{W}{1+\eta M} & -\beta M - \gamma W - \mu_G & -J_3 - \beta G_2 & \phi\frac{G_1}{1+\eta M} - \gamma G_2 \\ \varepsilon\beta M \left(1 - \frac{M+W}{K}\right) & \varepsilon\beta M \left(1 - \frac{M+W}{K}\right) & \varepsilon\beta \left(G_1 + G_2\right) J_1 - \mu_M & -\frac{\varepsilon\beta}{K}\left(G_1 + G_2\right) M \\ 0 & \delta\gamma W \left(1 - \frac{M+W}{K}\right) & -\frac{\delta\gamma}{K} W G_2 & \delta\gamma G_2 J_2 - \mu_W \end{bmatrix} \tag{19}$$

evaluated at the equilibrium point $P = (\bar{G}_1, \bar{G}_2, \bar{M}, \bar{W})$, where $J_1 = 1 - (2M + W)/K$, $J_2 = 1 - (M + 2W)/K$, and $J_3 = \phi G_1 \eta W / (1 + \eta M)^2$.

When two strains are competing for the same nutrient produced by only one of them, we have three equilibrium points: extinction (1), survival of the mutant strain only (2), and coexistence of both strains (2).

(1) Extinction of both populations – P^0 From equation (7), it is easy to verify that when $M = 0$ and $W = 0$, $G_1 = G_2 = 0$. This is the trivial (extinction) equilibrium point $P^0 = (0, 0, 0, 0)$. The Jacobian matrix J given by equation (8) evaluated at the trivial equilibrium point P^0 is

$$J^0 = \begin{bmatrix} -\mu_G & 0 & \alpha L_0 & 0 \\ 0 & -\mu_G & 0 & 0 \\ 0 & 0 & -\mu_M & 0 \\ 0 & 0 & 0 & -\mu_W \end{bmatrix},$$

which has the corresponding eigen-values $\lambda_1 = \lambda_2 = -\mu_G$, $\lambda_2 = -\mu_M$ and $\lambda_3 = -\mu_W$. Hence, P^0 is locally asymptotically stable (LAS) [1].

(2) Only the PCM survives – P^M If $W = 0$ (then $G_2 = 0$) and $M \neq 0$, the system of equations (17) becomes

$$\begin{cases} \frac{d}{dt}G_1 &= \alpha L_0 M - \mu_G G_1 - \beta G_1 M \\ \frac{d}{dt}M &= \varepsilon\beta G_1 M \left(1 - \frac{M+W}{K}\right) - \mu_M M, \end{cases}$$

and we have two positive equilibrium points $P^M_- = (\bar{G}_{1-}, 0, \bar{M}_-, 0)$ and $P^M_+ = (\bar{G}_{1+}, 0, \bar{M}_+, 0)$. This system of equations was analysed in the previous section. Briefly, when $\varepsilon\alpha L_0 < \mu_M$ there is only P^0 which is LAS; however when $\varepsilon\alpha L_0 > \mu_M$, if $\beta < \beta_0(\nu = 1)$ there is only P^0 which is LAS, while if $\beta > \beta_0(\nu = 1)$ there are three equilibrium points, where two equilibrium points P^0 and P^M_+ are LAS, while P^M_- is unstable. In this case, P^M_- divides the attracting regions of P^0 and P^M_+.

(3) Coexistence of both populations – P^* From equation (18), the equilibrium corresponding to the coexistence of mutant and wild-type strains $P^* = (\bar{G}_1, \bar{G}_2, \bar{M}, \bar{W})$ has

$$\begin{cases} \bar{M} = \dfrac{\left(\mu_G \frac{\chi_W}{\chi_M} + \gamma K\right)\chi_W \bar{G}_2 - \gamma K}{\alpha L_0 \chi_W - \left(\beta\frac{\chi_W}{\chi_M} - \gamma\right)\chi_W \bar{G}_2} \\ \bar{W} = \left(1 - \dfrac{1}{\chi_W \bar{G}_2}\right) - \bar{M} \\ \bar{G}_1 = \left(\dfrac{\chi_W}{\chi_M} - 1\right)\bar{G}_2, \end{cases}$$

where χ_M and χ_W are the fitness of mutant and wild strains defined by

$$\begin{cases} \chi_M &= \dfrac{\varepsilon\beta}{\mu_M} \\ \chi_W &= \dfrac{\delta\gamma}{\mu_W}, \end{cases}$$

and $\bar{G}_2$ is the positive root(s) of a third degree polynomial $P^3(G)$ given by

$$P^3(G) = p_3 G^3 + p_2 G^2 + p_1 G + p_0,$$

with the coefficients being given by

$$\begin{cases} p_3 &= ch^2 - (d+e)\,jh + gh^2 \\ p_2 &= -bo - 2coh + \left[(d+e)\,(jo + h\gamma K) + fjh\right] - 2gj\gamma K \\ p_1 &= 2boh + co^2 - \left[(d+e)\,o\gamma K + f\,(jo + h\gamma K)\right] + g\,(\gamma K)^2 \\ p_0 &= o\,(f\gamma K - bo)\,, \end{cases}$$

where

$$\begin{cases} a = \phi\left(\frac{\chi_W}{\chi_M} - 1\right) - \gamma & f = \eta\gamma K \\ b = aK & g = \eta\,(\gamma - \beta) \\ c = b - \mu_G & h = \beta\frac{\chi_W}{\chi_M} - \gamma \\ d = a + \beta & j = \mu_G\frac{\chi_W}{\chi_M} + \gamma K \\ e = \eta\,(\gamma K + \mu_G) & o = \alpha L_0 \chi_W. \end{cases}$$

Notice that $\bar{G}_1 > 0$, and both mutant and wild strains coexist if

$$\chi_W > \chi_M. \tag{20}$$

In other words, the wild-type strain must have better fitness to survive and coexist with the mutant strain; otherwise ($\chi_M \geqslant \chi_W$) the wild-type strain cannot survive.

The coefficient p_0 can be written as a second degree polynomial for γK,

$$p_0\,(\gamma K) = o\left[\eta\,(\gamma K)^2 + \alpha L_0 \chi_W\,(\gamma K) - \alpha L_0 \chi_W \phi\left(\frac{\chi_W}{\chi_M} - 1\right)\right],$$

which has two roots $(\gamma K)_- < 0$ and $(\gamma K)_+ > 0$. The polynomial is such that $p_0\,(\gamma K) < 0$, for $\gamma K < (\gamma K)_+$, and $p_0\,(\gamma K) > 0$, for $\gamma K > (\gamma K)_+$. The coefficients p_3, p_2 and p_1 can be analysed to determine the number of positive roots of the polynomial $P^3(G)$. The behaviour of the system of equations (17) is determined by the number of the equilibrium points P^*, which is given by the number of positive roots of $P^3(G)$ as described elsewhere [2, 3].

References

[1] Yang, H. M. (2014). The basic reproduction number obtained from Jacobian and next generation matrices–A case study of dengue transmission modelling. Biosystems, 126, 52-75.

[2] Delboni, R. R.; Yang, H. M. Mathematical Model of Interaction Between Bacteriocin-producing Lactic Acid Bacteria and Listeria. Part 1: Steady States and Thresholds. B. Math. Biol., v. 79, p. 1637-1661, 2017.

[3] Delboni, R. R.; Yang, H. M. Mathematical Model of Interaction Between Bacteriocin-producing Lactic Acid Bacteria and Listeria. Part 2: Bifurcations and Applications. B. Math. Biol., v. 79 (10), p. 2273-2301, 2017.
